# Sources of bias and limitations of thrombinography: inner filter effect and substrate depletion at the edge of failure algorithm

**DOI:** 10.1186/s12959-023-00549-5

**Published:** 2023-10-04

**Authors:** Joseph W. Jackson, Colin Longstaff, Samuel A. Woodle, William C. Chang, Mikhail V. Ovanesov

**Affiliations:** 1 Center for Biologics Evaluation and Research, U.S. Food and Drug Administration, 10903 New Hampshire Ave, Silver Spring, MD 20993-0002 United States of America; 2 National Institute for Biological Standards and Control (NIBSC), Potters Bar, Hertfordshire, UK; 3 Present Address: Walter Reed National Military Medical Center, 4301 Jones Bridge Rd, Bethesda, MD 20814 United States of America; 4 Present Address: The Henry M. Jackson Foundation for the Advancement of Military Medicine, Inc., Walter Reed Army Institute of Research, 503 Robert Grant Ave, Silver Spring, MD 20910 United States of America

**Keywords:** Thrombin, Blood coagulation test, Factor VIII, Hemophilia A, Hemostasis

## Abstract

**Background:**

Fluorogenic thrombin generation (TG) is a global hemostasis assay that provides an overall representation of hemostasis potential. However, the accurate detection of thrombin activity in plasma may be affected by artifacts inherent to the assay-associated fluorogenic substrate. The significance of the fluorogenic artifacts or their corrections has not been studied in hemophilia treatment applications.

**Methods:**

We sought to investigate TG in hemophilia plasma samples under typical and worst-case fluorogenic artifact conditions and assess the performance of artifact correction algorithms. Severe hemophilic plasma with or without added Factor VIII (FVIII) was evaluated using commercially available and in-house TG reagents, instruments, and software packages. The inner filter effect (IFE) was induced by spiking elevated amounts of fluorophore 7-amino-4-methylcoumarin (AMC) into plasma prior to the TG experiment. Substrate consumption was modeled by adding decreasing amounts of Z-Gly-Gly-Arg-AMC (ZGGR-AMC) to plasma or performing TG in antithrombin deficient plasma.

**Results:**

All algorithms corrected the AMC-induced IFE and antithrombin-deficiency induced substrate consumption up to a certain level of either artifact (edge of failure) upon which TG results were not returned or overestimated. TG values in FVIII deficient (FVIII-DP) or supplemented plasma were affected similarly. Normalization of FVIII-DP resulted in a more accurate correction of substrate artifacts than algorithmic methods.

**Conclusions:**

Correction algorithms may be effective in situations of moderate fluorogenic substrate artifacts inherent to highly procoagulant samples, but correction may not be required under typical conditions for hemophilia treatment studies if TG parameters can be normalized to a reference plasma sample.

**Supplementary Information:**

The online version contains supplementary material available at 10.1186/s12959-023-00549-5.

## Introduction

Accurate laboratory assessment of hemostasis potential is needed to diagnose and guide treatment of bleeding and prothrombotic conditions. Clinical laboratories screen for major bleeding defects with the relatively quick and affordable tests of prothrombin time (PT) and activated partial thromboplastin time (aPTT). Rare coagulation pathologies caused by individual coagulation factor or plasma inhibitor deficiencies are diagnosed with an extensive panel of tests that measure plasma antigen or activity levels of each coagulation protein, one protein at a time. However, the complexity and redundancy of the coagulation cascade all but assures that the relative deficiency or elevation of one protein will not always translate into an increased or decreased coagulation potential of patient plasma.

Discovered in the 1950s, evaluation of thrombin generation (TG) has been employed with variable success to assess overall hemostasis and abnormal coagulability of blood and plasma samples [1]. The TG assay remained difficult for clinical laboratories until Hemker et al. simplified it with the help of high throughput microtiter plates, chromogenic substrate for thrombin, and automated analysis software [2]. Modern commercial TG assays employ a fluorogenic substrate to thrombin, which yields advantages over chromogenic substrates by removing the interference of turbidity in analyzing samples; unlike the chromogenic predecessor, the fluorescent substrate is not substantially encumbered by fibrin, platelets, and other materials that may interfere with the TG assay readout, ultimately allowing for increased sample diversity [3].

Fluorescence based TG readouts are subject to the unique artifacts of their own that can affect the assay results. Some of the artifacts are plasma sample-specific, e.g., the effect of a sample’s optical density on the fluorescence signal. Other artifacts are inherent to the nature of synthetic TG substrates. For example, substrate consumption can cause a reduction of signal (substrate consumption rate), and therefore the recorded activity of thrombin can be underestimated. Fluorophore-based substrates are also prone to an inner filter effect (IFE), which is a phenomenon where the fluorescence response is suppressed and deviates from linearity at higher concentrations of fluorophore. The IFE is caused by re-absorption of the emitted light by the fluorophore that ultimately reduces the signal that is visible to the detector. One group of TG assays, based on Hemker’s calibrated automated thrombogram (CAT) algorithm, accounts for many substrate artifacts by comparing TG in plasma samples to wells with a reference thrombin activity calibrator, a thrombin-α_2_ macroglobulin complex (Tα_2_MG), capable of cleaving the substrate in the absence of actual TG in the plasma sample [3].

Independent of fluorescent artifacts, use of high amounts of thrombin-specific synthetic substrates can also disrupt the balance of natural TG reactions. Butenas and Mann [4] demonstrated this by measuring tissue factor (TF)-induced TG in the presence or absence of the most commonly used thrombin fluorogenic substrate, Z-Gly-Gly-Arg 7-amino-4-methylcoumarin (ZGGR-AMC); this substrate delayed the initiation time and the time to reach thrombin peak, and increased the thrombin peak height (TPH). These changes can be explained by the competition between the synthetic fluorogenic substrate, which acts as a reversible inhibitor for thrombin, plasma coagulation factors (native substrates for thrombin), and natural irreversible inhibitors for thrombin.

Substrate artifacts do not rule out the clinical potential of TG assays. For example, the competition with the synthetic substrate does not necessarily prevent the detection of differences in TG between healthy and pathological samples [4]. Similarly, our previous investigations suggested that the IFE and substrate consumption can be of little consequence for detection of elevated TG in normal plasma spiked with increasing levels of coagulation factors I, V, VIII, IX, X, and XI [5]. On the other hand, algorithmic CAT corrections for substrate consumption were helpful in moderate cases of elevated prothrombin [6] and antithrombin deficiency [7]. Yet, in more severe conditions of substantially elevated prothrombin or deficient antithrombin, CAT algorithm failed to produce TG curve parameters due to its inability to process substantially distorted fluorescence signals.

In the current work, we designed experiments to delineate the limits on the algorithmic corrections, by delivering a predictable degree of IFE and substrate consumption. Our goal was to find the conditions where the artifact distortions are so strong that they are no longer correctable by CAT algorithm, i.e., at the edge of algorithm failure.

**Fig. 1:**
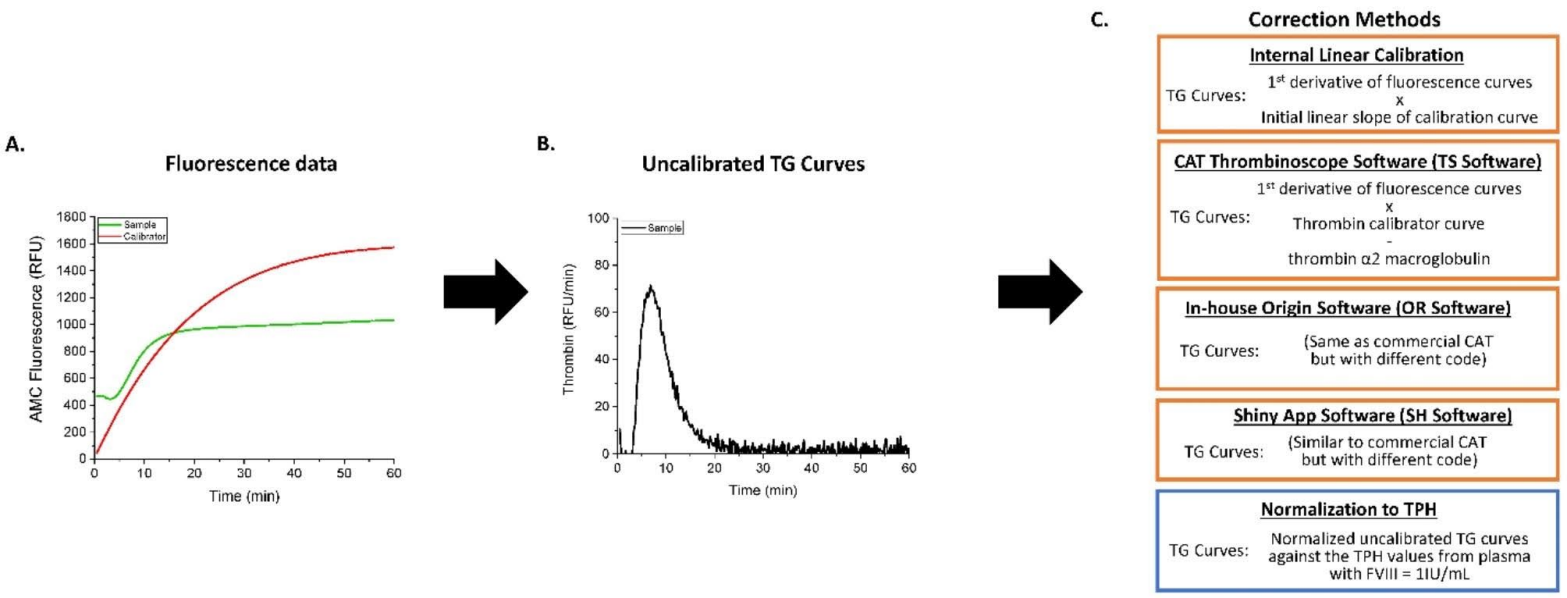
Schematic of thrombin generation curve correction methods. This schematic is to describe the correction methods for thrombin generation (TG) and outline these different algorithms used in this study. **(A)** Fluorescence curves for plasma samples (green) and for calibrator wells (red) are recorded. **(B)** The first derivative of these fluorescence curves is taken to generate uncalibrated TG curves in relative fluorescence units (RFU) per minute (RFU/min), and **(C)** subsequently corrected in the indicated calibration methods: Internal Linear Calibration (1st derivate of the fluorescence curves, i.e., uncalibrated curves, are multiplied by the initial linear slope of the calibration curve; provides calibration without fluorescence artifact correction), TS Software (i.e., commercial CAT Thrombinoscope software; 1st derivate is multiplied by the thrombin calibration curve with thrombin-α_2_ macroglobulin signal values subtracted), OR software (i.e., our in-house version of CAT coded in Origin LabTalk software), and SH software (a web-based interface written by one of us (CL) which performs calibrations and corrections in a similar way to the CAT software), see materials and Methods. A normalization method was also employed such that uncalibrated curves were normalized against the normal thrombin peak height (TPH) values measured in normalized plasma samples with added FVIII (i.e., FVIII-DP + 1 IU/mL FVIII).

Edge of failure, a concept from the control of manufacturing processes, describes the conditions where the investigated complex process stops behaving in an acceptable fashion (i.e., fails in achieving the desired outcomes) due to technical limitations such as non-linear effects [8, 9]. Edge of failure analysis can define the risks of the system failing to achieve target performance when operated under challenging conditions. Here, we sought to elucidate the impact of the substrate artifacts, and qualify the corrective benefits of the CAT calibrator, at the edge of TG assay failure for hemophilia A plasma samples with normalized FVIII concentration, as well as antithrombin deficient plasma samples. We employed three versions of the CAT correction algorithm (Fig. 1), commercial Thrombinoscope software (denoted here as TS software) [3], our in-house app for TG analysis written in LabTalk language for Origin statistical and graphical software (Origin; denoted as OR software) [5, 6, 10], as well as the publicly available software, labelled SH, developed by one of us (CL) as part of a project to improve reproducibility and transparency supported by the International Society for Thrombosis and Haemostasis (ISTH) Subcommittee on Fibrinolysis [11].

We have achieved algorithm failure after substrate consumption by extremely procoagulant samples or extreme IFE when two of the correction software packages failed to correct for the most disfigured TG curves. Below the edge of failure, calibration correction was efficient in restoring the TG curve shape. Interestingly, artifacts did not disrupt the ability of the assay to distinguish between our normal and pathological samples despite the IFE and substrate consumption effects on both samples.

## Materials and methods

### Materials

The fluorophores 7-amino-4-methylcoumarin (AMC) and 7-amino-4-trifluoromethylcoumarin (AFC) and fluorogenic substrate Z-Gly-Gly-Arg-AMC (ZGGR-AMC) were from Bachem (Bubendorf, Switzerland). Fluorogenic substrate ZGGR-AFC was from AnaSpec Inc. (Fremont, CA, USA). Tris BSA buffer, pH 7.4 (Biophen chromogenic substrate buffer) was from Aniara Diagnostica (West Chester, OH, USA). FVIII-deficient frozen plasma (FVIII-DP) from a single individual with severe hemophilia A (FVIII < 1%) was from HRF, Inc. (Raleigh, NC, USA). Affinity depleted antithrombin-deficient frozen plasma (ATIII-DP) and normal pooled frozen plasma (VisuCon) were from Affinity Biologics (Ancaster, ON, Canada). Corn trypsin inhibitor (CTI) and rabbit thrombomodulin were from Haematologic Technologies, Inc. (Essex Junction, VT, USA). Commercially available recombinant tissue plasminogen activator (tPA) and plasma-derived Antihemophilic Factor/von Willebrand Factor Complex (Human) concentrate (denoted as FVIII concentrate) were purchased as drugs from the NIH pharmacy in Bethesda, MD. Calibrated Automated Thrombinography (CAT) reagents TF with lipid vesicles (PPP-Low), fluorogenic substrate ZGGR-AMC (Fluo-Substrate), calcium chloride buffer (Fluo-Buffer), and thrombin activity calibrator (Tα2MG complex with a known substrate cleaving activity expressed as thrombin concentration) were from Stago USA (Parsippany, NJ, USA). In ATIII-DP experiments, we used recombinant lipidated TF (HemosIL RecombiPlasTin 2G) from Instrumentation Laboratory (Bedford, MA, USA), procoagulant phospholipid vesicles (Rossix Phospholipid-TGT) from Diapharma (West Chester Township, OH, USA), and heparin and calcium chloride from Sigma Aldrich (St. Louis, MO, USA).

### AMC and AFC spectra

Excitation and emission recording was conducted on a Synergy H4 microplate reader (Biotek, Winooski, VT, USA) regulated at 37 °C in a narrow bandwidth (9.0 nm) excitation and emission monochromator mode.

### CAT TG assay

CAT assay was performed according to the manufacturer’s recommendations (80 µL plasma, 20 µL PPP trigger or 20 µL thrombin calibrator, and 20 µL FluCa) with some modifications described below. Briefly, in the unmodified CAT protocol, FVIII-DP was supplemented with a final concentration of 1 IU/mL of FVIII or an equal volume of buffer instead of FVIII (plasma:buffer volume ratio, 49:1); plasma was placed in a microplate (Immulon HB, Stago) and mixed with PPP-Low reagent. The experiment was then initiated by injection of the mixture of provided Fluo-Substrate and Fluo-Buffer (i.e., FluCa reagent mixture) using a Fluoroskan Ascent microplate reader (Stago). To allow for thrombin calibration and artifact correction, two plasma wells were supplemented with CAT thrombin calibrator in place of PPP-low.

For experiments where we sought to elucidate the severity of the IFE impact on TG results, the IFE was simulated by spiking 40 to 200 µM of fluorophore AMC into plasma (plasma:AMC volume ratio, 4:1) prior to the TG experiment (equivalent to a ~ 10 to ~ 50% pre-consumption of fluorogenic substrate).

For substrate consumption experiments, plasma was supplemented with the appropriate amount of pre-diluted ZGGR-AMC substrate (Bachem; plasma:substrate volume ratio, 4:1), after which the PPP-Low and calcium chloride reagents were mixed with half of the plasma wells. The experiment started with the injection of thrombin calibrator (with the help of the injector inside the Fluoroskan Ascent microplate reader) into the remaining half of the wells (to serve as calibrator wells).

For substrate consumption experiments in the presence of two fluorogenic substrates, CAT protocol was modified to allow for the simultaneous recalcification of multiple wells as follows: pre-diluted mixtures of ZGGR-AMC (Bachem) with or without ZGGR-AFC in calcium chloride (to a total concentration of 800 µM for the sum of both substrates) were arranged on the bottom of a 96 well assay microplate (Immulon HB); plasma was mixed with PPP-Low or thrombin calibrator reagents in another microplate; a 96-channel pipettor (Matrix Hydra DT, Thermo Fisher Scientific, Waltham, MA, USA) transferred plasma-reagent mixture to wells containing substrate and calcium chloride mixture, thereby initiating the reaction. Data recording was conducted on a Synergy H4 microplate reader at 37^o^C in a monochromator mode at 380 nm excitation and 430 nm emission (both with 9 nm bandwidth) every 52 s.

### In-house TG/FG/FL assay for procoagulant samples

To investigate the procoagulant samples, modeled with a mixture of ATIII-DP and normal plasma, we used a TG, fibrin generation and fibrinolysis (TG/FG/FL) assay performed as described previously [12] with minor modifications. Briefly, ATIII-DP was supplemented with 5% normal pooled plasma, CTI (100 µg/mL), rabbit thrombomodulin (12.5 nM), tPA (0.13 µg/mL), synthetic PC:PS vesicles (4 µM), ZGGR-AMC (800 µM) and TF (titrated from 0.12–20 pM, diluted in Tris-BSA buffer) or thrombin calibrator in the presence or absence of heparin (0.03 USP units/mL). A 96-channel pipettor (Viaflo 96, Integra Biosciences, Hudson, NH, USA) was used to transfer the plasma mixture to a half-area 96-well plate containing fluorogenic substrate ZGGR-AMC (Bachem) with calcium chloride. Recording was conducted on a Synergy H4 at 37^o^C by two channels in a fast filter mode, a fluorescent channel (360 nm excitation and 460 nm emission, both with 40 nm bandwidth) and absorbance channel (490 nm), every 24 s per a two-channel cycle.

### CAT algorithm processing software

The software apps (see Fig. 1) used in this study were: commercial CAT software Thrombinoscope ver. 5.0.0.742 (denoted as TS software), an in-house LabTalk script package based on Origin software (denoted as OR software; Origin Lab, Northampton, MA, USA), which is available upon request and described previously [5, 10], and the publicly available online app written in the R language [13] with the Shiny web interface package [14] (denoted as SH software). Links to the working SH software, detailed help notes, and code are available via [15]. TS software was used for all experiments on the CAT instrument. Additionally, our OR software and SH software were used for all experiments, including the analysis of raw data generated from CAT. Although the TG analysis can return multiple TG curve parameters, our previous investigations suggested that thrombin peak height (TPH) and endogenous thrombin potential (ETP; measured as area under the TG curve) are affected the most by the IFE [5].

Little is known about the curve fitting, smoothing and baseline handling by the TS software. The differences between the SH and OR software packages are in (1) the fitting of the thrombin calibrator curve which is used for CAT correction during processing, i.e. the 3rd order polynomial curve fitting in OR software and the 4th order polynomial in SH software, and (2) the use of preprogrammed first derivative algorithms as available in Origin and R language, specifically, the use of Origin 2 point Savitzky-Golay smooth derivative (OR software) and TG curve smoothing with the Friedman’s SuperSmoother R language function (SH software).

### Normalization approach

To allow for the comparison of uncalibrated and calibrated TG results in FVIII deficient plasma, we performed a normalization of TPH values in hemophilia A plasma on the values in plasma supplemented with 1 IU/mL of FVIII. Normalization was performed for TPH values derived both from the uncorrected TG curves as well as the TG curves reported by TS, OR, or SH software apps.

### Internal linear calibration of thrombin activity without CAT corrections

To compare the CAT algorithm output with the TG curves calibrated in the absence of IFE and substrate consumption corrections, we used an internal linear calibration curve approach using a single calibration coefficient (i.e., the same coefficient was used regardless of fluorescence level) as described previously [5].

### Edge of failure analysis

Edge of failure analysis of forced artifact effect experiments (i.e., experiments with spiked AMC, reduced substrate, or reduced antithrombin conditions) was performed to identify the edge of failure set points, determined as the failure of a given software app to calculate a TG curve.

### Statistical analyses

Statistical analyses were performed in Origin Pro 2020. The mean and SD are reported, where possible. The difference between the means was assessed with a two-sample t-test at indicated significance levels (0.05, as reported). Results of analysis and the associated 95% confidence intervals are summarized in the text.

## Results

### The Inner Filter Effect

**Fig. 2:**
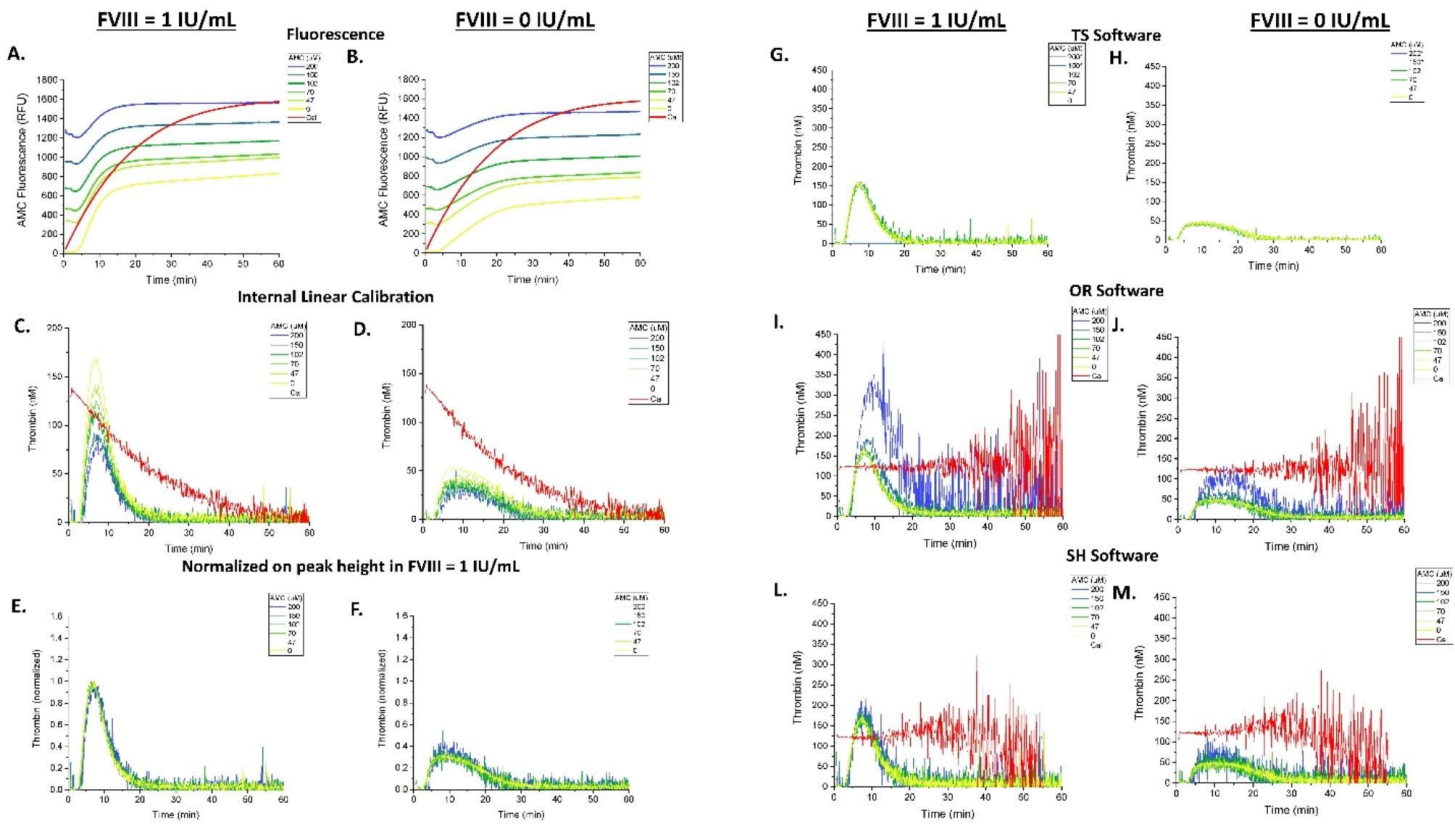
IFE of AMC fluorophore and its correction via calibration or normalization. FVIII-DP was supplemented with 1 IU/mL FVIII to normalize hemophilia plasma, or not, and was subsequently premixed with the indicated concentrations of AMC prior to initiating coagulation with Ca^2+^ and substrate. Raw fluorescent data were produced by the CAT microplate reader and software and analyzed in several different ways: **(A, B)** raw AMC fluorescence in relative fluorescent units (RFU), **(C, D)** internally calibrated TG curves via a thrombin calibration coefficient (see Materials and Methods), **(E, F)** normalized-uncalibrated curves, **(G, H)** calibrated TG curves (via TS software), **(I, J)** calibrated TG curves (via OR software), and **(L, M)** calibrated TG curves (via SH software). Uncalibrated curve data were produced by differentiating the AMC curves observed in **(A, B)**. Calibrated curves were produced using TS software, our in-house OR software, which uses published algorithms similar to CAT calibration, or SH software, our second in-house app based on CAT algorithm. An asterisk (*) next to the indicated concentrations in panels G & H denotes high AMC concentrations in which commercial TS software did not report TG curves, possibly due to their noisy appearance as suggested by the TG curves reported by OR and SH software apps at these high concentrations. Normalized-uncalibrated curves were produced by normalizing each uncalibrated curve pairing of hemophilic sample and normalized hemophilia sample (hemophilic plasma supplemented with FVIII) at each pre-spiked AMC concentration against the TPH value of the normalized plasma sample in each pairing. TG was recorded for 40–60 min. Assay conditions: 63 µL of FVIII-DP, 1 µL of FVIII (1 IU/mL), 16 µL of AMC at indicated concentrations, 20 µL of PPP trigger, and 20 µL of FluCa.

To test whether the IFE non-linearity can disrupt the TG assay, plasma samples were spiked with increasing concentrations of fluorophore AMC (0 to 200 µM) prior to triggering TG (Fig. 2). The resulting fluorescence curves showed a baseline fluorescence intensity proportional to AMC concentration in both hemophilia and FVIII supplemented plasma samples (Fig. 2A,B). An unexpected artifact of fluorescence signal drop was observed within the first 5 min on each fluorescence curve, which corresponded to a negative substrate consumption rate. It is possible that these momentary changes in fluorescence signal are indicative of the settling of plasma-calcium mixtures following the injection of calcium chloride into AMC-containing plasma. This artifact was also observed in experiments without added substrate (compare supplemental Fig. S1A and Fig. S1B), suggesting that the drop is unrelated to the IFE under investigation in this experiment, but is likely caused by the optical changes in plasma absorbance after addition of DMSO (AMC diluent).

Without correction for IFE and substrate consumption (i.e. only differentiation of the fluorescence curve and internal linear thrombin calibration, see Materials and Methods), the resulting TG curves showed an inverse, concentration-dependent effect of pre-spiked fluorophore on the general size of the curve (Fig. 2C,D). We normalized the uncalibrated TG curves at each pre-spiked AMC concentration against the TPH value of the corresponding sample of hemophilia plasma supplemented with FVIII (Fig. 2E,F). This method produced overlapping normalized TG curves for all TG conditions, confirming that AMC did not change the TG curves but only induced the IFE.

**Fig. 3:**
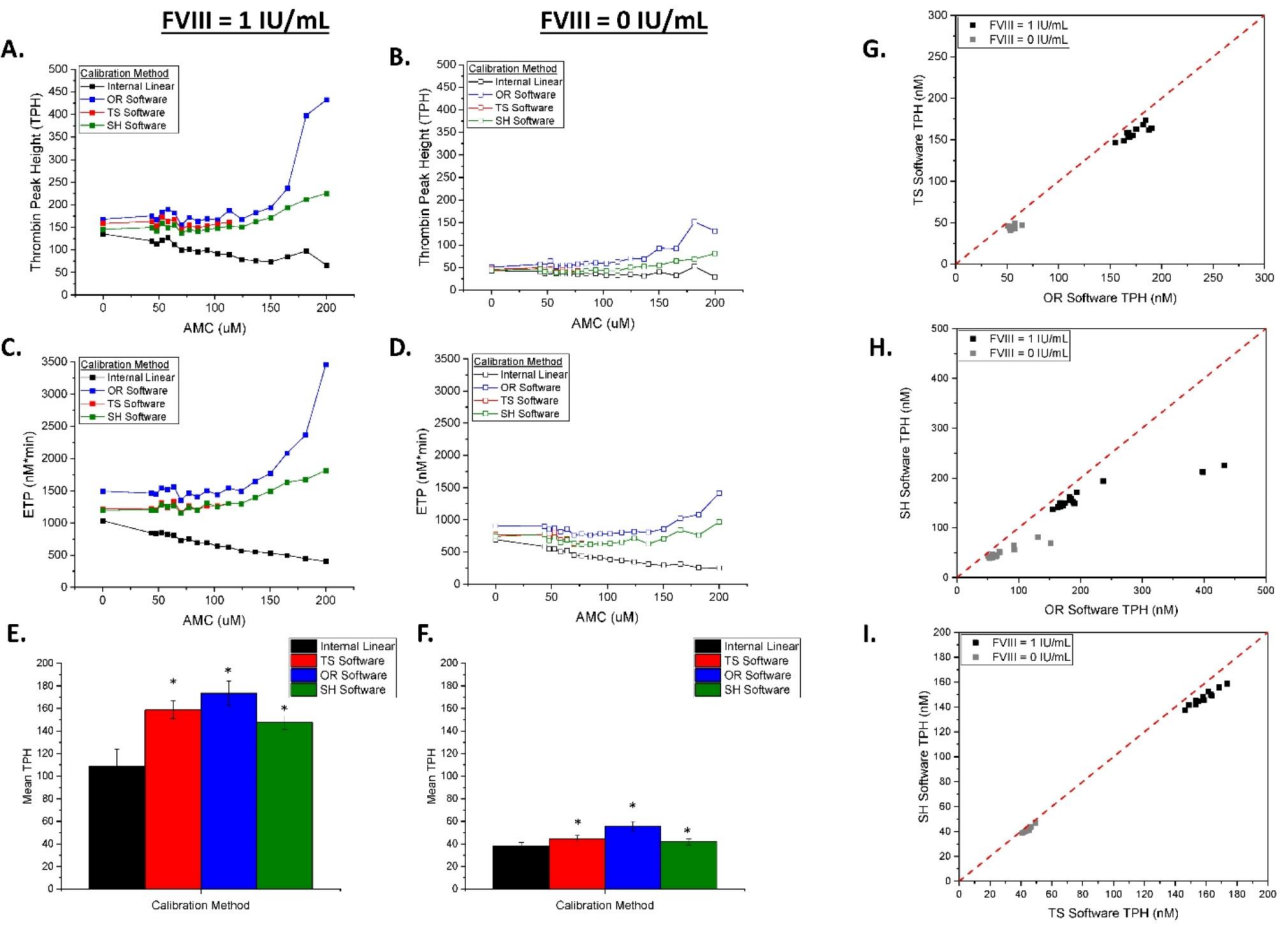
CAT calibration algorithm distorts TG curves when the fluorescence signal extends into the non-linear range of the calibrator. Thrombin peak height (TPH) and endogenous thrombin potential (ETP; measured as area under the curve) values were obtained from data in Fig. 1. TPH values from indicated software apps in **(A)** FVIII-DP supplemented with 1 IU/mL FVIII and **(B)** FVIII-DP with added buffer. ETP values from indicated software apps in **(C)** FVIII-DP supplemented with 1 IU/mL FVIII and **(D)** FVIII-DP with added buffer. Internal linear calibration is shown as black curves, CAT calibration is shown as blue curves (OR software), red curves (TS software), and green curves (SH software). CAT calibration correction did not return values at AMC concentrations > 115 µM. **(E)** TPH values from FVIII-DP supplemented with 1 IU/mL FVIII and **(F)** FVIII-DP with added buffer in indicated software apps, where p < 0.05 was deemed significantly different via a paired T-test (denoted as *) as compared to the Internal Linear group. Correlations of TPH from **(G)** TS software vs. OR Software, **(H)** SH software vs. OR Software, and **(I)** SH software vs. TS software in plasma with 0 IU/mL FVIII (gray squares) and with 1 IU/mL FVIIII (black squares). Assay conditions: 63 µL of FVIII-DP, 1 µL of FVIII (1 IU/mL), 16 µL of AMC at indicated concentrations, 20 µL of PPP trigger, and 20 µL of FluCa.

The TG curves were reanalyzed to test the algorithmic correction of IFE. TS software produced overlapping curves at AMC concentrations below 115 µM (Fig. 2G,H; and Fig. 3); no TG curve data (curve = 0) were reported for experiments with AMC above 115 µM or 75 µM in samples with either 1 IU/mL FVIII or no added FVIII, respectively (Fig. 3A-D). When OR software was used for corrections analysis, the resulting TG curves appeared to overlap except at the highest concentrations of spiked AMC fluorophore, where they were erratic (Fig. 2I,J). Correction via SH software produced overlapping TG curves, with small overestimation at the highest AMC concentrations (Fig. 2L,M).

To investigate the reasons behind the overestimation of TG curves by OR and SH apps at high AMC concentrations, we plotted thrombin calibrator curves before (red lines in Fig. 2C-D) and after CAT algorithm corrections (red lines Fig. 2I-M) by OR and SH software. TS software app data were excluded from this analysis because TS does not report thrombin calibrator data. As expected, a sharp decline in thrombin activity was observed immediately after the beginning of the experiment (Fig. 2C-D), demonstrating the effects of IFE and substrate consumption in the TG assay. This decline was accurately resolved by CAT algorithm in the OR software, as evidenced by the same average thrombin calibrator activity at a horizontal line position of red calibrator curves in Fig. 2I-J. However, an increasing level of distorting noise was seen after 30 min, reflecting the amplification of noise in the uncalibrated curves in Fig. 2C-D. In contrast, SH software maintained accurate linearization for a period of 20 min only, followed by an overestimation of the thrombin calibrator activity from 20 to 30 min and progressive underestimation after 40 min (red lines in Fig. 2L-M). These differences in accuracy of corrective linearization of the thrombin calibrator curve correlated with the degree of overestimation of TG curves in AMC-supplemented experiments, i.e., underestimation of thrombin calibrator activity by SH in the area of extreme non-linearity of the calibrator curve (above 40 min, see red lines in Fig. 2A-B) resulted in less extreme overestimation of TG curves at the highest AMC spiked concentrations.

Figure 3 shows the TG assay parameters TPH and ETP as a function of AMC concentration. Since AMC fluorophore reagent does not interfere with the reaction of substrate cleavage by thrombin, the algorithms were expected to report comparable thrombin activities for either AMC concentration. Under- or over-estimation of reported activity indicates that the algorithm, as implemented in a particular software package, will over- or undershoot its correction, respectively. The OR software was able to accurately recover both TPH and ETP up to AMC fluorophore concentrations of ~ 170 µM, overestimating TPH (Fig. 3A,B) and ETP (Fig. 3C,D blue lines) at higher AMC. The TS software produced similar values for both parameters, except when the software did not compute the TG parameters for FVIII-supplemented plasma above 115 µM of spiked AMC (Fig. 3A,C) and hemophilic plasma samples above 75 µM of spiked AMC (Fig. 3B,D, red lines). For both plasma samples tested, SH software was able to recover both TPH and ETP at all AMC concentrations, however, a trend to overestimation was apparent above 115 µM of AMC (Fig. 3A-D). The mean TPH values for the indicated correction algorithms were significantly different when compared to values produced by internal linear calibration in both plasma samples (Fig. 3E,F). A correlation between the three studied apps is shown in Fig. 3G. The side-by-side comparisons between the three software outputs (Fig. 3A-D) indicated similar curve shapes. Additionally, the curves did not fully overlap. Further investigation suggested that these differences can be due to the smoothing applied by the TS software to the calibrated TG curves prior to parameter acquisition (refer to Supplemental Fig. S2 for additional evidence of smoothing).

Overall, calibration by the CAT method, as implemented in the TS, OR and SH software apps, demonstrated adequate correction of suppressed rate of substrate consumption due to IFE that was induced by added baseline fluorescence. This correction was accurate in recovering correct thrombin activity when the fluorescence values were within the linear range of the thrombin calibrator wells. At higher fluorescence (above ~ 115 µM AMC, Table 1), calibration by TS or OR software apps eventually reached the edge of failure, either failing to produce TG results (TS software) or overestimating the TG parameters (OR software). SH software recovered TG parameters at all AMC concentrations. Interestingly, normalization to TPH in FVIII-supplemented plasma successfully produced overlapping TG curves, that where much less erratic than the curves produced by TS or OR software, suggesting another unintended consequence of correction algorithm, i.e. amplification of noise. This amplification was more apparent at higher concentrations AMC, consistent with the stronger IFE induced by AMC and stronger corrections applied by the algorithm.

**Table 1:** Edge of failure set points for fluorescent artifacts on TG analysis. The significance the IFE and substrate consumption on TG as analyzed by three software packages: TS, OR, and SH

Identified Edge of Failure^¶^					
Fluorescence Artifact	Edge of Failure Experiment	Thrombinoscope (TS) Software	In-house ORIGIN (OR) Software	CL Shiny App (SH Software)	TPH Normalization Method
**IFE**	FVIII-DP spiked with [AMC]: 0, 47, 70, 102, 200 µM	**FVIII = 1 IU/mL**: Corrected at [AMC] < 115 µM **FVIII = 0 IU/mL**: Corrected [AMC] < 75 µM	**FVIII = 1 IU/mL**: Overestimated results at [AMC] > 170 µM **FVIII = 0 IU/mL**: Corrected [AMC] > 170 µM	**FVIII = 1 IU/mL**: Successful at all AMC concentrations **FVIII = 0 IU/mL**: Successful at all AMC concentrations	**FVIII = 1 IU/mL**: Successful at all spiked [AMC] **FVIII = 0 IU/mL**: Successful at all spiked [AMC]
Substrate Consumption*	Reduced ZGGR-AMC at range: 0-836 µM	n/a*†	n/a*	n/a*	n/a*
	Reduced ratio of ZGGR-AFC supplemented to ZGGR-AMC ZGGR-AFC: 0, 200, 400, 600, 700, 750, 800 µM	n/a*†	n/a*	n/a*	n/a*
**Procoagulant Samples**	ATIII-DP ± heparin with range of TF concentrations successfully induced substrate consumption	n/a†	Not corrected at any TF concentration	Successfully corrected at all TF concentrations	Corrected at all TF concentrations but lag time was modulated

¶ Edge of failure is defined as the failure of CAT correction algorithms to calculate a TG curve

* Substrate consumption was never fully achieved most likely due to Tα_2_MG activity

† These experiments were performed on a Biotek Synergy plate reader, and thus commercial CAT/TS Software experiments were not recorded

### Effect of reduced substrate concentration

**Fig. 4:**
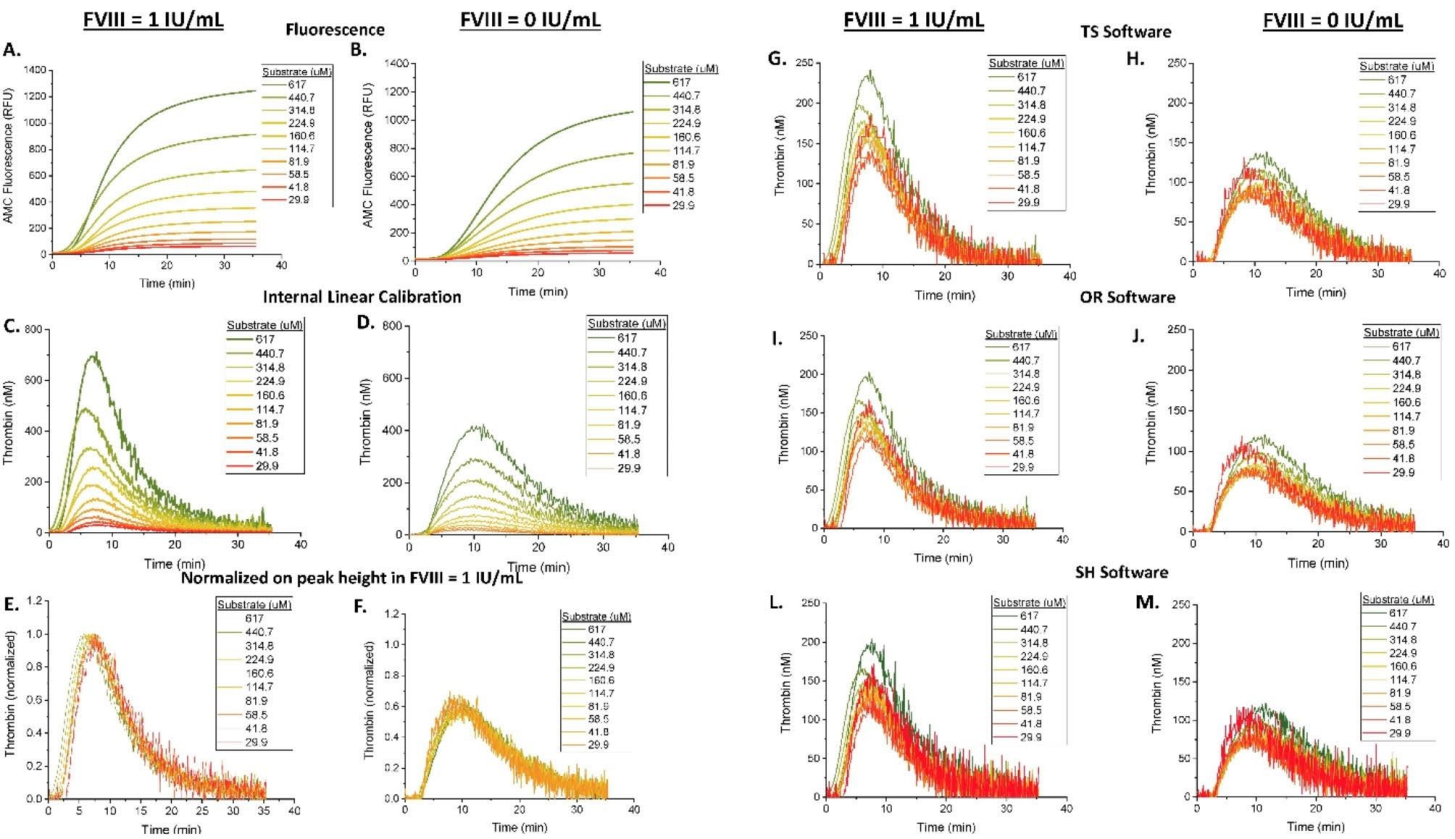
Substrate consumption and its correction via calibration or normalization. FVIII-DP was supplemented with 1 IU/mL FVIII to normalize hemophilic plasma, or not, and was subsequently premixed with the indicated concentrations of substrate, ZGGR-AMC, prior to initiating coagulation with Ca^2+^ and substrate. Raw data was produced by the CAT microplate reader and analyzed in several different ways: **(A, B)** raw AMC fluorescence in relative fluorescent units (RFU), **(C, D)** internally calibrated TG curves via a thrombin calibration coefficient (see Materials and Methods), **(E, F)** normalized-uncalibrated curves, **(G, H)** calibrated TG curves (via TS software), **(I, J)** calibrated TG curves (via OR software), and **(L, M)** calibrated TG curves (via SH software). Uncalibrated curves data were produced by differentiating the AMC curves observed in **(A, B)**. Calibrated curves were produced using TS, OR or SH apps all of which employed the same CAT correction algorithm. Normalized-uncalibrated curves were produced by normalizing each uncalibrated curve pairing of hemophilic and normalized sample (hemophilic plasma supplemented with FVIII) at each pre-spiked AMC concentration against the TPH value of the normalized plasma sample in each pairing. TG was recorded for 40 min. Assay conditions: 63 µL of FVIII-DP, 1 µL of FVIII (1 IU/mL), 16 µL of substrate ZGGR-AMC (at indicated concentrations), 20 µL of PPP trigger, and 20 µL of Ca^2+^ (calcium chloride buffer)

We next sought to investigate the corrective capabilities of algorithmic calibration in conditions of substrate depletion. Indeed, complete substrate consumption would be characterized by a fluorescence increase rate reaching zero value at a certain time point, leading to no TG activity recorded regardless of the CAT artifact correction algorithm. To facilitate substrate depletion, plasma samples were supplemented with decreasing concentrations of fluorogenic substrate (Fig. 4). The fluorescent curves showed a concentration-dependent decrease in overall fluorescent intensity as substrate concentration decreased (Fig. 4A,B). As expected, internal linear calibration without CAT correction resulted in TG curves that were decreased with lower substrate concentrations (Fig. 4C,D).

The CAT algorithm (in all three software packages) was able to reconstruct TG curves at all tested substrate concentrations without reaching the point of failure, suggesting that complete substrate consumption was not observed under any tested condition in Fig. 4. Another indication of incomplete substrate consumption was that normalization to TPH (in FVIII-supplemented plasma) appeared to correct for the substrate concentration effects with great consistency for all samples, producing overlapped curves, suggesting that the shape of TG curve was not affected by substrate concentrations (Fig. 4E,F). Faster onset of substrate consumption would have changed the shape of TG curve. However, normalized TG curves appeared distorted by noise at low substrate concentrations, likely because reduced signal (in samples with reduced substrate concentration) to noise ratio resulted in more erratic curves.

### Interference of substrate with TG reactions

Interestingly, in substrate titration experiments, all three software packages produced an overestimation of TG curves at the highest substrate concentration (Fig. 4I-M). Previous studies suggested that fluorogenic substrate can interfere with TG kinetics. Therefore, decreasing the initial substrate concentration, which is used at a concentration of 416 µM in commercial assays and 800 µM in our laboratory, may have impacted the TG kinetics independently of, and in addition to, the artifact of substrate depletion. To address the effect of substrate on TG reactions, we used another ZGGR substrate based on an AFC fluorophore rather than AMC. A mixture of AFC and AMC substrates can maintain the total concentration of ZGGR peptide allowing us to investigate the effect of AMC-based substrate consumption without adverse manipulation of TG kinetics. We assumed that the specificity of ZGRR substrate to thrombin and FXa and other enzymes has not changed substantially as a result of replacing the AMC fluorophore with the AFC fluorophore.

We confirmed that AFC has peak fluorescent emission shifted to the right of that of AMC (~ 490 nm vs. ~450 nm; Supplemental Fig. S3A,B). Unfortunately, in the CAT instrument the AFC fluorophore alone produced a much higher signal than that of AMC, because the CAT microplate reader uses a filter set favored by the AFC, i.e., the 390 nm excitation is closer to preferred AFC excitation than that of AMC and the 460 nm emission is close enough to AFC emission peak (see Supplemental Fig. S4). Therefore, we used a monochromator microplate reader (Biotek) in a narrow-band mode of 380 nm excitation and 430 nm emission (shifted lower than CAT instrument’s wavelengths). A control experiment on this microplate reader using an AMC-only substrate titration under the same conditions and reagents as used for the CAT experiment demonstrated consistent results between CAT and Biotek readers (compare Fig. 4 to Supplemental Fig. S5). TS software was not able to use fluorescent data produced by Biotek reader, and therefore only OR and SH software were compared below.

**Fig. 5:**
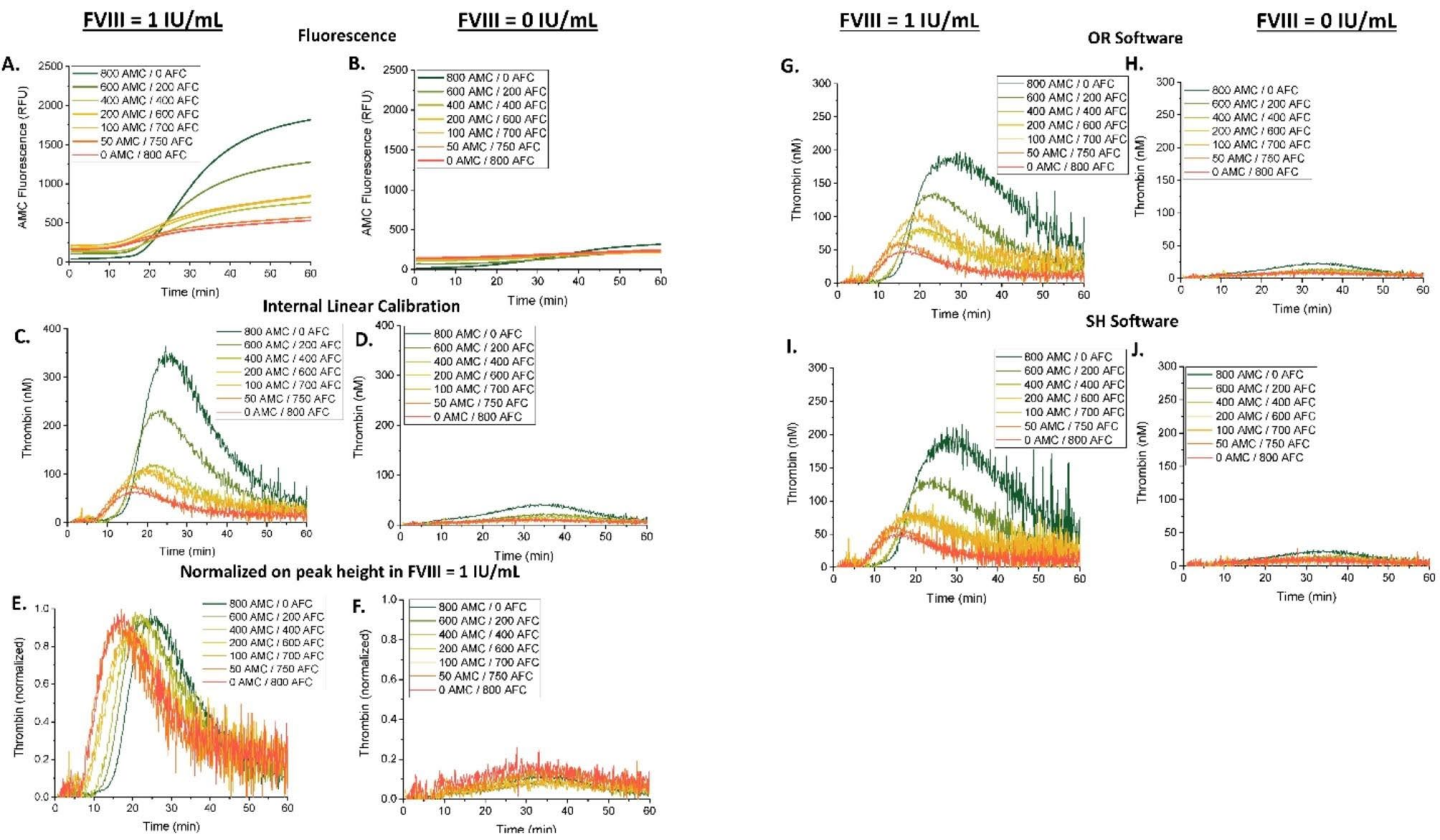
An attempt to study substrate consumption in plasma samples supplemented with two substrates, ZGGR-AMC and ZGGR-AFC. FVIII-DP was supplemented with 1 IU/mL FVIII to normalize hemophilic plasma, or not, and was subsequently premixed with the indicated concentrations of two substrates, ZGGR-AMC and ZGGR-AFC, such that the ratio of AMC:AFC equaled to a concentration of 800 µM, prior to initiating coagulation with Ca^2+^. Raw data was produced by the Biotek microplate reader and analyzed in several different ways: **(A, B)** raw AMC fluorescence in relative fluorescent units (RFU), **(C, D)** internally calibrated TG curves via a thrombin calibration coefficient (see Materials and Methods), **(E, F)** Normalized-Uncalibrated curves, **(G, H)** Calibrated TG curves (via OR software), and **(I, J)** calibrated TG curves (via SH software). Uncalibrated curve data were produced by differentiating the AMC curves observed in **(A, B)**. CAT calibrated curves were produced using our in-house OR and SH software apps, which use published algorithms similar to CAT calibration. Normalized-uncalibrated curves were produced by normalizing each uncalibrated curve pairing of hemophilic and normalized sample (hemophilic plasma supplemented with FVIII) at each pre-spiked AMC concentration against the TPH value of the normalized plasma sample in each pairing. TG was recorded for 40–60 min. An artifact resembling TG signal in early minutes in Fig. 5 is only seen with ZGGR-AFC experiments, suggesting that it is caused by either the AFC fluorophore itself (similar to Fig. 2 and Fig. S1 discussed above) or background fluorescence signal of un-cleaved ZGG-AFC substrate. Assay conditions: 78 µL of FVIII-DP, 2 µL of FVIII (1 IU/mL), 20 µL of PPP trigger, and 20 µL of custom FluCa mixtures (substrates ZGGR-AMC and ZGGR-AFC at indicated concentrations with calcium chloride buffer)

Because the AMC/AFC substrate mixture experiments contained the same combined concentration of ZGGR peptide at the beginning of the experiment, the samples within each hemophilic plasma pair (with and without 1 IU/mL FVIII) were expected to have the same TG reactions regardless of the concentration of added ZGGR-AMC substrate. In contrast, recording of the TG curves were predicted to be affected by the consumption of AMC substrate. As expected, a decrease in the substrate consumption rate (Fig. 5A,B) was observed with decreasing concentrations of AMC substrate.

Similarly to AMC substrate titration experiments above (Fig. 4), the AMC/AFC substrate mixture experiments showed that the AMC substrate was not completely consumed in either plasma sample group, as evidenced by the continuous sloped increase at the tail end of the fluorescent curves (Fig. 5A,B), which suggests that residual substrate cleaving activity (likely that of the thrombin-α_2_ macroglobulin complex formed at early stages of the TG curve) was continuing to cleave substrate. Therefore, an edge of failure caused by complete substrate depletion did not occur in hemophilic plasma with and without added FVIII regardless of the substrate concentration (Table 1). However, CAT correction algorithm failed to alleviate the underestimation of the TG in samples with lower than typical substrate concentrations, see Fig. 5G,H (OR software) and Fig. 5I,J (SH software). Normalization of the TG curves to the TPH values produced curves that were slighlty more overlapped and less erratic than the calibration method (Fig. 5E,F). It should be noted, however, that both the normalization and CAT methods produced TG curves with higher lag times as the concentration of ZGGR-AMC increased, preventing a full overlap of TG curves.

Interestingly, similar to experiments with spiked AMC, the baseline fluorescence intensity of each sample increased as AFC substrate concentration increased, suggesting that ZGGR-AFC substrate produced a baseline fluorescent signal even before it was cleaved, despite employing a wavelength set by the Biotek plate reader (Fig. 5A,B). This bleed-through of the ZGGR-AFC and possible AFC fluorescence into the AMC recording channel is the likely reason for the failure of CAT algorithm to correct for AMC substrate consumption in the AMC/AFC substrate mixing experiments.

### Complete substrate consumption by Procoagulant samples

Previous TG investigations suggested that complete substrate depletion can be achieved in procoagulant conditions caused by deficiency of antithrombin [5, 7]. In another attempt to produce forced controlled substrate depletion, we used moderately antithrombin deficient plasma (ATIII-DP, 5% deficiency) with or without a dose of heparin that partially compensated for antithrombin deficiency (by heparin-mediated increase in antithrombin activity). Further, to determine the edge of failure for the variable substrate consumption conditions, we produced dose-dependent elevation of TG curves with increasing concentrations of TF trigger.

**Fig. 6:**
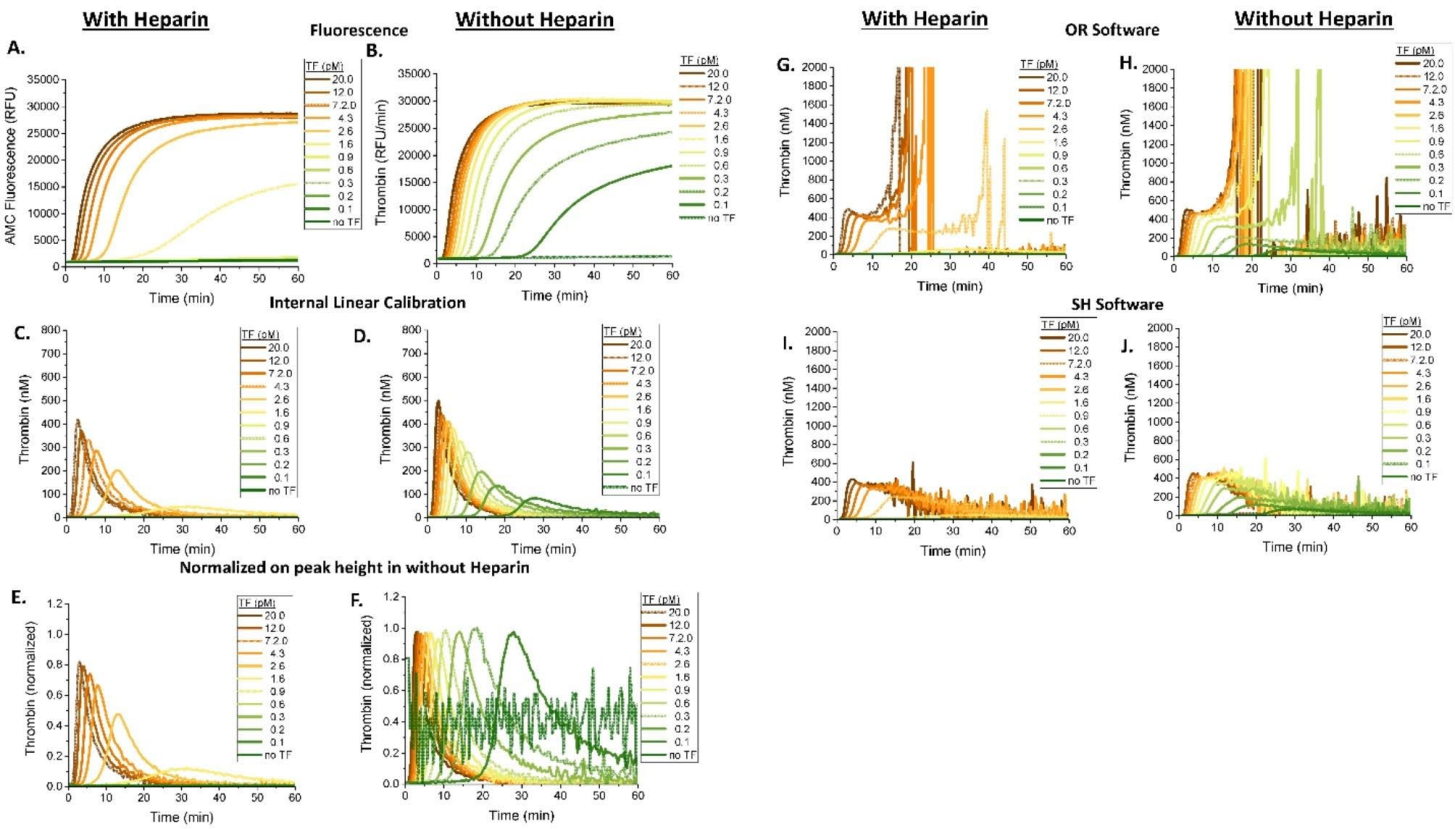
Effect of CAT calibration on TG curves in procoagulant plasma samples. Antithrombin deficient plasma (ATIII-DP) was treated with or without heparin and the indicated TF concentration to assess the effect of calibration via different software apps in procoagulant samples. Raw data was produced by the CAT assay microplate reader and analyzed in several different ways: **(A, B)** raw AMC fluorescence in relative fluorescent units (RFU), **(C, D)** internally calibrated TG curves via a thrombin calibration coefficient (see Materials and Methods), **(E, F)** normalized-Uncalibrated curves, **(G, H)** calibrated TG curves (via CBER algorithm), and **(I, J)** calibrated TG curves (via SH software). Uncalibrated curve data were produced by differentiating the AMC curves observed in **(A, B)**. CAT calibrated curves were produced by our in-house OR and SH software apps. Normalized-uncalibrated curves were produced by normalizing each uncalibrated curve pairing of hemophilic and normalized sample (hemophilic plasma supplemented with FVIII) at each pre-spiked AMC concentration against the TPH value of the normalized plasma sample in each pairing. TG was recorded for 60 min. Assay conditions: ATIII-DP with 0.2 U/mL of normal pooled plasma with or without heparin (0.03 USP/mL), TF (0.12–20 pM), tPA (0.13 µg/mL), thrombomodulin (12.5 nM), PC:PS vesicles (4 µM) and custom FluCa mixture (800 µM ZGGR-AMC and calcium chloride)

As expected, AMC fluorescence curves were dose dependent on TF concentration (Fig. 6A,B). Internal linear calibration without CAT corrections demonstrated dose dependent TG curves, with higher and quicker curves generated at high TF concentrations (Fig. 6C,D). CAT algorithm with the OR software produced highly distorted TG curves comprised of the initial elevation and peak reminiscent of the beginning portion of the uncalibrated TG curved, yet with no decline in thrombin activity following the peak (Fig. 6G,H). Instead, TG peaks were followed by a brief plateau of thrombin concentration and another sharp elevation into infinite values. The second TG peak in OR software was caused by an artifact of thrombin activity overestimation, suggesting algorithm failure following complete depletion of the substrate, which was seen previously in our study of TG at elevated prothrombin levels [5, 7]. In contrast, SH software produced curves that were much less erratic but had plateau-like thrombin peaks between 20 and 4.3 pM of TF (Fig. 6I,J). Again, less overstimation by SH than OR software was consistent with its underestimation of linear calibrator concentrations closer to the end of the calibration curve (similar to red lines in Fig. 2L-M).

Consistent with previous observations in procoagulant samples with complete substrate depletion [5, 7], OR and SH software apps produced TG curves with a highly erractic tails (portion of the curve after the TG peak) in ATIII-DP samples, with higher noise as TF concentration increased. Erratic increases in TG curve tails are not likely to represent sudden spikes in thrombin activity, but rather an effect of amplification of fluorescence curve noise which is caused by an overshooting of the CAT algorithm corrections at small bumps in uncalibrated TG curve tails. This overshooting represents a failure of CAT algorithm to accurately reconstruct TG curves from miniscule growth of the fluorescence signal when fluorescence growth is hindered by substrate depletion [5, 7].

To further evaluate if exaggerated TG tails are an artifact of CAT algorithm, we studied clot formation and lysis through observation of clot turbidity in parallel with the TG experiments described above (see Supplemental Fig. S6). As expected, times to clot formation aligned well with the beginning of the TG curves (Fig. S6) and were inversely correlated with TPH. Further, the peak clot density was inversely correlated with TPH, and clot density peaked at a TF concentration of ~ 2.6 pM in the presence of heparin, whereas in the samples without heparin, clot density was highest at very low TF concentrations (~ 0.1 pM). Since clot formation is limited by the depletion of fibrinogen, clot density may not accurately reflect the TG curves. Yet, increased TG should translate into protection from tPA-induced fibrinolysis as we reported previously in this experimental system [12]. Nonetheless, time to clot lysis appeared to (inversely) correlate with the TPH calculated from the first clear peak on the TG curve (see Fig. S6). This is likely explained by the fact that no reactions of fibrinolysis begin before fibrin formation. When the lysis time is corrected for the clot time, the duration of clot lysis does correlate positively with the TF dose. Overall, monotonous changes in clotting and lysis parameters corresponded to monotonous changes in TPH parameter rather than apparently erratic changes of TG curve tail ends.

## Discussion

The use of TG assays in assessing hemostasis has potential in both pre-clinical and clinical applications. The modern method for TG assays includes the use of a fluorogenic substrate that is cleaved by thrombin to produce a readout of thrombin activity. Some commercial TG assays further employ a correction algorithm, commonly known as CAT approach, to account for the artifacts of using fluorescent reagents. The two most cited artifacts, IFE and substrate consumption, are thought to produce TG results that underestimate actual thrombin activity. Here, we sought to investigate the corrective ability of CAT algorithm using the commercial software app and two publicly available alternatives, our in-house Origin and R/Shiny software apps [13–15]. Algorithmically-processed curves were compared with uncalibrated curves and a linear calibration (that does not account for substrate artifacts [5], yet uses initial rates measured in thrombin calibrator wells to recalculate relative fluorescence into thrombin activity), as well as to normalization of pathological samples on “normal” plasma conditions (see Fig. 1). These comparisons were done under specially designed forced conditions where the artifacts of IFE and substrate consumption were intentionally extreme or exaggerated.

In the data presented here, we have demonstrated that the sample normalization on TPH of the reference plasma (i.e., the comparison of FVIII-DP to FVIII-DP supplemented with 1U/mL FVIII), which is a model of the approach used previously [16], corrects for such artifacts to various degrees, usually in a more robust manner than the software apps that employ the CAT artifact correction algorithm. Unfortunately, this simple normalization approach is only possible when both the reference plasma and the tested sample are affected equally by the artifact, e.g., when substrate concentration is decreased equally for both plasma samples. Under this equally affected requirement, calibrator reagents and mathematical processing appeared unnecessary even with extreme artifacts that could bring CAT algorithm to the “edge of failure”. Assessing the integrity of these corrective methods for TG in cases where substrate artifacts are extreme can highlight the strengths and limitations for CAT analysis under specific sample conditions, such as when the substrate consumption is increased in highly procoagulant plasma samples [7].

The extent to which the fluorogenic substrate artifacts may influence the diagnostic utility of the TG assay remains unresolved. To some degree, interference of the assay conditions with natural coagulation processes is inherent to any in vitro assay of hemostasis. For example, substantial dilution of blood or plasma is known to affect the balance of procoagulant and anticoagulant reactions, yet traditional one stage clotting or chromogenic substrate factor activity assays currently rely on sample dilution with large volumes of coagulation triggers and other assay reagents. Indeed, use of large reagent volumes is necessary to ensure accurate dispensing and adequate mixing which are extremely important for robustness of hemostasis assays. Likewise, in TG methods, the artifacts of synthetic substrates may be of little consequence to how well the assay distinguishes between normal and abnormal samples and have an added benefit of increased signal-to-noise ratio due to increased thrombin signal in the presence of substrate. The substrate for thrombin acts like its reversible inhibitor, therefore, the substrate reagent is blocking inhibitory action of natural thrombin inhibitors antithrombin and fibrinogen, increasing the fluorescent signal generated by the active thrombin.

Our data confirmed that IFE and substrate consumption introduce clear assay non-linearity, which is evidenced from the sharply declining activity in the thrombin calibrator wells (red lines in Fig. 2C-D), and that CAT algorithm is capable of linearization of this effect, albeit at the expense of fluorescence noise amplification (Fig. 2I-J). Interestingly, some commercially available and widely used TG platforms do not use CAT algorithm to correct for these or any other fluorogenic substrate artifacts. Furthermore, even for TG systems that utilize CAT algorithm, these corrections can address only the fluorescence artifacts without correcting for the interference of synthetic substrate with coagulation reactions, such as inhibition of free thrombin (due to the Tα_2_M complex calibrator not binding to antithrombin).

Our investigation of dosed forced delivery of fluorescence artifacts shows that the CAT correction may indeed be helpful at moderate degrees of the artifactual distortion and for some TG parameters, but that available correction algorithms have limitations, which manifest in failures to correct intensely strong distortions. Extreme IFE in the form of supplemented AMC (Fig. 2) or substrate depletion in antithrombin deficient samples (Fig. 6) produced extreme noise amplification as well as the underestimation of TG curves. The most affected TG parameter was ETP due to the necessity of the entire length of TG curve for the ETP measurement. However, two of the three software packages were successful for ETP correction only at relatively low degree of IFE distortion (Fig. 3). Correction algorithms failed to produce accurate ETP in samples with extreme conditions of high IFE or very procoagulant samples with high substrate consumption. The TPH parameter was less affected because it does not require the entire length of TG curve, and thus the TPH appears to serve as a more consistent parameter for TG measurement in the presence of moderate IFE or substrate consumption. In more extreme artifact samples, the commercial TS app failed to quantify either the ETP or TPH parameters, highlighting the limitations of the software. It is likely that TS software app errs on the side of caution by refusing to report any data for the extremely distorted TG curves.

Our elucidation of the edge of failure set points for conditions aggravated by the IFE artifact for hemophilic samples has demonstrated that the TS and OR software failed to properly correct for IFE in the extreme non-linear range of the assay, whereas we obtained adequate results when comparing hemophilic plasma normalized to control plasma. Normalized TG was relatively insensitive to the IFE, as added AMC concentrations of 115 µM caused only ~ 20% modulation in TPH, whereas the TS software could not produce TG curves at this level of induced IFE. Thus, in the case of hemophilic plasmas and hemophilic plasmas with supplemented FVIII, comparison to normalized TG parameters may return useful TG information even without artifact correction.

Similar to our previous works that used spiked normal plasma samples, we observed that substrate consumption is not an artifact that will affect results unless a truly procoagulant sample is being measured. Complete substrate consumption was seen in the procoagulant conditions of antithrombin deficiency, and this depletion failed to be fully corrected by the correction algorithm (Fig. 6). Corrections in antithrombin deficiency returned erratic TG tails, preventing the accurate estimation of the ETP and TPH values. Interestingly, the edge of failure was never established in our substrate-deficient conditions used to simulate substrate depletion (see Figs. 4 and 5), suggesting that the CAT algorithm failure in highly procoagulant conditions is not related to substrate consumption per se, as the assay handles extremely low levels of substrate without failure. Rather, our CAT algorithm failures were apparently caused by the combination of extreme non-linearity of high fluorescence signal (likely caused by IFE) and the readout noise, as seen in experiments with spiked AMC or antithrombin deficient plasma. These results are consistent with our previous observations that correction of substrate artifacts is not necessary in many procoagulant samples, with the notable exceptions of conditions when high fluorescence signal is accumulated due to fast substrate consumption (but not necessarily substrate depletion) in elevated prothrombin [5] and antithrombin deficiency [5] conditions.

A parallel can be drawn between fluorogenic substrate consumption (in TG experiments) and fibrinogen consumption (a natural substrate of thrombin during the clotting process). Because clottable fibrinogen is depleted within minutes of coagulation, clot turbidity curves are not suitable for the detection of the full TG curve, due to not being able to show absorbance changes soon after the clot was formed and before the peak of TG is reached. Nonetheless, we observed lower optical density readouts at increasing TG, suggesting that clotting assays can carry some information about the TG peak. It was suggested that more intense coagulation and thrombin formation induced by the higher TF concentrations ultimately results in lower optical density readouts, likely because higher thrombin produces thinner fibrin fibres which have lower optical density [17].

Our results agree well with the previously published approach to standardize TG assay through normalization on normal reference plasma [16]. In this work we observed the TG assay ability to distinguish between clinically significant phenotypes of hemophilic plasma vs. FVIII-supplemented plasma depending on induced artifacts. Normalizing the TG results to the TPH values of the FVIII-DP supplemented with 1 IU/mL FVIII successfully corrected the TG curves affected by reduced substrate or induced IFE. Notably, a 20-fold decrease in substrate concentration did not significantly change the shape of the curves (except at the highest substrate concentrations, which produced a slightly different shape in either sample) nor did it change the ratio of hemophilic to normalized plasma, suggesting that the presence of synthetic substrate should not diminish the assay’s power to separate normal from hemophilic samples. These results also suggest that normalization on reference plasma may be helpful when artifacts are expected to affect both samples. Previously, the effect of FVIII on TG was shown to be independent of another TG reagent, TF [18]. Specifically, van Veen et al. investigated the effect of both 1 pM and 5 pM TF concentrations on TG parameters in plasma from normal, mild, and severe hemophilia patients. The authors concluded that the concentration of TF did not affect the ability of CAT analysis to distinguish between normal and hemophilic samples [18]. Further, the authors mentioned that the standardization of the TG assay is prevented in part due to the absence of an international reference standard to TF and the absence of a TG reference plasma sample. Again, a method to normalize plasma samples to that of a reference plasma could provide a suitable way to measure TG in hemophilic plasmas.

Our study has important limitations. We did not study various advanced artifact correcting algorithms [19] other than the ones included in the traditional CAT approach as published by Hemker et al. [3]. Nor we aimed to investigate sample-specific fluorescence artifacts, such as the effect of inter-individual variability of plasma sample’s optical effect on the fluorescent signal. This was due to our using the same plasma source (FVIII-DP or ATIII-DP) supplemented or not with the source of deficient protein and thus the variability among multiple patient samples could not be inferred. Further, our simulated samples, i.e., supplemented commercial deficient plasmas, are not expected to fully reflect observations made from plasma samples derived from patients. For the purposes of this study, FVIII-DP samples supplemented or not with FVIII were affected equally by our artificially induced IFE experiments; real-life clinical samples that present different procoagulant levels will need to be tested if they are affected differently by the IFE and substrate artifacts. Thus, future experiments on patient samples will further delineate the effects of IFE and substrate consumption in samples derived from different patient donors.

## Conclusions

TG correction algorithms may be effective in situations of moderate fluorogenic substrate artifacts inherent to highly procoagulant samples. However, correction may not be required under typical conditions for replacement hemophilia treatment studies if TG parameters can be normalized to a reference plasma sample.

### Electronic supplementary material

Below is the link to the electronic supplementary material.

Supplementary Material 1 Fig. S1. Fluorescence artifacts within first 5 minutes of experiment observed in the absence and presence of added fluorogenic substrate for thrombin. AMC fluorescence in plasma samples: (**A**) without added substrate and (**B**) with added substrate (concentration indicated on respective lines). Red curves denote FVIII-DP and black curves denote FVIII-DP with added 1 IU/mL FVIII. These artifacts explain “negative” thrombin activity early in TG curves on Fig. 1, as we observe an increase in fluorescence despite the lack of added substrate (panel A).

Supplementary Material 2 Fig. S2: Differences in CAT and Origin software correction algorithm. The differences of the CAT and Origin software (CBER) calibration methods may be due to additional CAT-proprietary smoothing algorithms, which are applied to (calibrated) TG curves prior to parameter acquisition. Shown is a representative screenshot of a CAT-calibrated TG curve. Highlighted in red boxes are the reported, calculated peak value of 163.73 nM, even though the TG curve itself shows a peak value of 182.9 nM.

Supplementary Material 3 Fig. S3: AMC and AFC fluorophore emission characteristics. The raw RFUs of the indicated concentrations of (**A**) AMC and (**B**) AFC were calculated and showed a proportional increase in fluorescence with increasing concentration. Emission (nm) of AMC was calculated at ~450 nm, whereas AFC was ~490 nm.

Supplementary Material 4 Fig. S4. Fluorescence of AMC and AFC fluorophores on the CAT microplate reader. The fluorescence of the indicated concentrations of AMC and AFC were measured on the CAT microplate reader. The AFC fluorophore gives a higher signal than that of AMC in CAT instrument demonstrating that this is not suitable for the substrate consumption experiments with added AMC and AFC (Fig. 4).

Supplementary Material 5 Fig. S5. Substrate supplementation on FVIII-DP samples. FVIII-DP was supplemented with 1 IU/mL FVIII to normalize plasma, or not, and was subsequently premixed with the indicated concentrations of AMC prior to initiating coagulation with Ca2+. Raw data was produced by the Biotek microplate reader and software and analyzed in several different ways: (**A, B**) raw AMC fluorescence in relative fluorescent units (RFU), (**C, D**) Externally calibrated TG curves via a thrombin calibration coefficient (see Materials and Methods), (**E, F**) Normalized-Uncalibrated curves, (**G, H**) Calibrated TG curves (via OR software and (**I, J**) Calibrated TG curves (via SH software). Uncalibrated curve data were produced by differentiating the AMC curves observed in (**A, B**). Calibrated curves were produced using our in-house OR software, which uses published algorithms similar to CAT calibration, or SH software. Normalized-uncalibrated curves were produced by normalizing each uncalibrated curve pairing of hemophilic and normalized sample (hemophilic plasma supplemented with FVIII) at each pre-spiked AMC concentration against the TPH value of the normalized plasma sample in each pairing. TG was recorded for 40-60 minutes.

Supplementary Material 6 Fig. S6. Clot formation and lysis in procoagulant samples. ATIII-DP was treated with the indicated concentrations of TF and treated (**A**) with heparin or (**D**) without heparin and clot formation was subsequently measured via a fibrin generation (FG) assay (see Materials and Methods). The corresponding correlations between (**B, E**) clot density (OD 490) vs. TPH (nM) and (**C, F**) time to clot (min) vs TPH (nM) were plotted.

Supplementary Material 7 Fig. S7. Calibration curves for experiments in Fig. 4. Calibration curves for the indicated substrate concentrations are shown: (**A**) Fluorescence curves, (**B**) Uncalibrated curves from calibrator wells, (**C**) calibration curves after Internal Linear calibration, (**D**) calibration curves after calibration via OR software, and (**E**) calibration curves after calibration via SH software.

Supplementary Material 8 Fig. S8. Calibration curves for experiments in Fig. 5. Calibration curves for the indicated AMC/AFC substrate concentrations are shown: (**A**) Fluorescence curves, (**B**) Uncalibrated curves from calibrator wells, (**C**) calibration curves after Internal Linear calibration, (**D**) calibration curves after calibration via OR software, and (**E**) calibration curves after calibration via SH software.

Supplementary Material 9 Fig. S9. Calibration curves for experiments in Fig. 6. Calibration curves in the presence (red curves) or absence (green curves) of heparin: (**A**) Fluorescence curves, (**B**) Uncalibrated curves from calibrator wells, (**C**) calibration curves after Internal Linear calibration, (**D**) calibration curves after calibration via OR software, and (**E**) calibration curves after calibration via SH software.

## Data Availability

All data generated or analysed during this study are included in this published article [and its supplementary information files].

## Abbreviations

a AMC: amino-4-methylcoumari
AFC: 7-amino-4-trifluoromethylcoumarin
ATIII(-DP): Antithrombin-(deficient plasma)
CAT: Calibrated Automated Thrombinography
ETP: Endogenous thrombin potential
FVIII(-DP): Coagulation Factor VIII-(deficient plasma)
IFE: Inner filter effect
OR: Origin LabTalk software
PT: Prothrombin time
SH: Shiny App software
Tα_2_MG: Thrombin-α_2_ macroglobulin
TG: Thrombin generation
TPH: Thrombin peak height
TS: Thrombinoscope software
ZGGR-AMC: Carbobenzoxy-Gly-Gly-7-amino-4-methylcoumarin
