# Supplementary figures and images for: Sources of bias and limitations of thrombinography: inner filter effect and substrate depletion at the edge of failure algorithm
Source: Thromb J. 2023 Oct 4;21:104. doi: 10.1186/s12959-023-00549-5 (PMC10548689; doi:10.1186/s12959-023-00549-5)

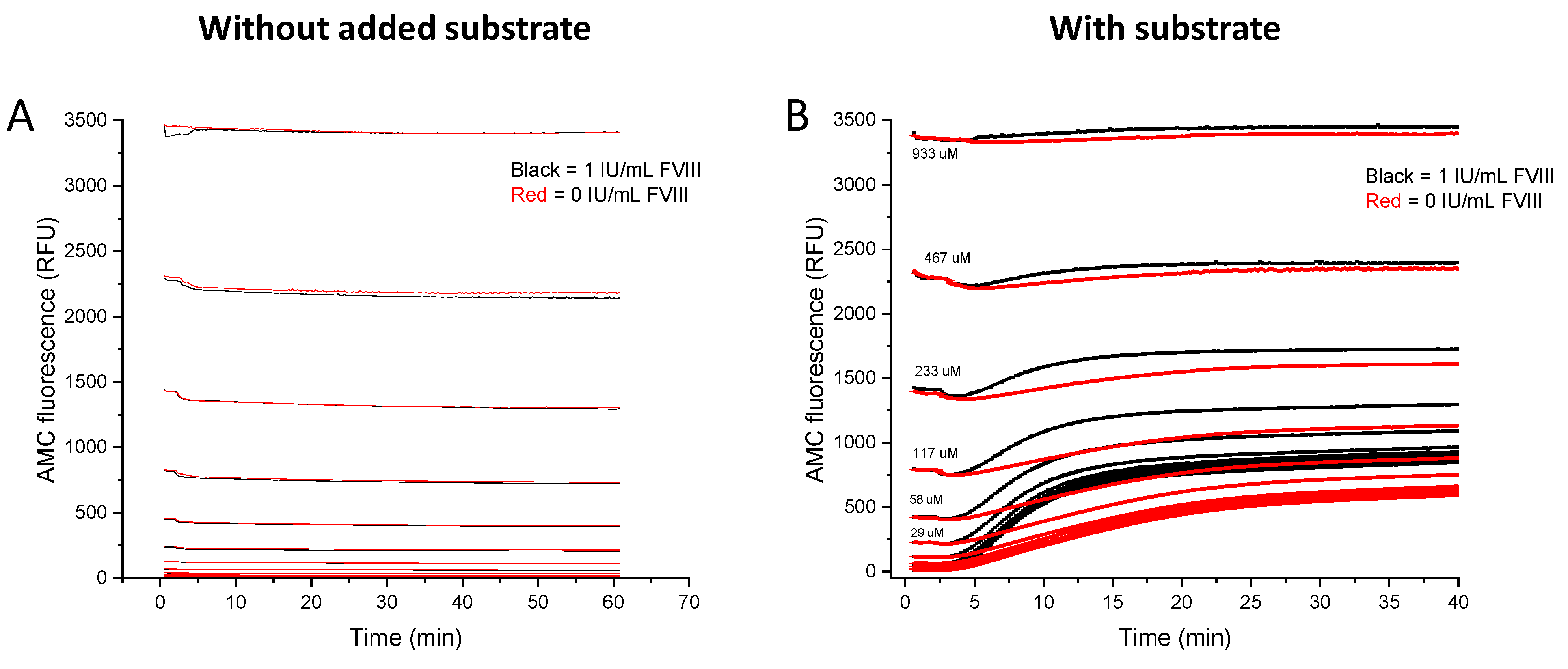

Supplement: Supplementary file 1 — Supplementary Material 1 Fig. S1. Fluorescence artifacts within first 5 minutes of experiment observed in the absence and presence of added fluorogenic substrate for thrombin. AMC fluorescence in plasma samples: (A) without added substrate and (B) with added substrate (concentration indicated on respective lines). Red curves denote FVIII-DP and black curves denote FVIII-DP with added 1 IU/mL FVIII. These artifacts explain “negative” thrombin activity early in TG curves on Fig. 1, as we observe an increase in fluorescence despite the lack of added substrate (panel A). [file 12959_2023_549_MOESM1_ESM.tiff]

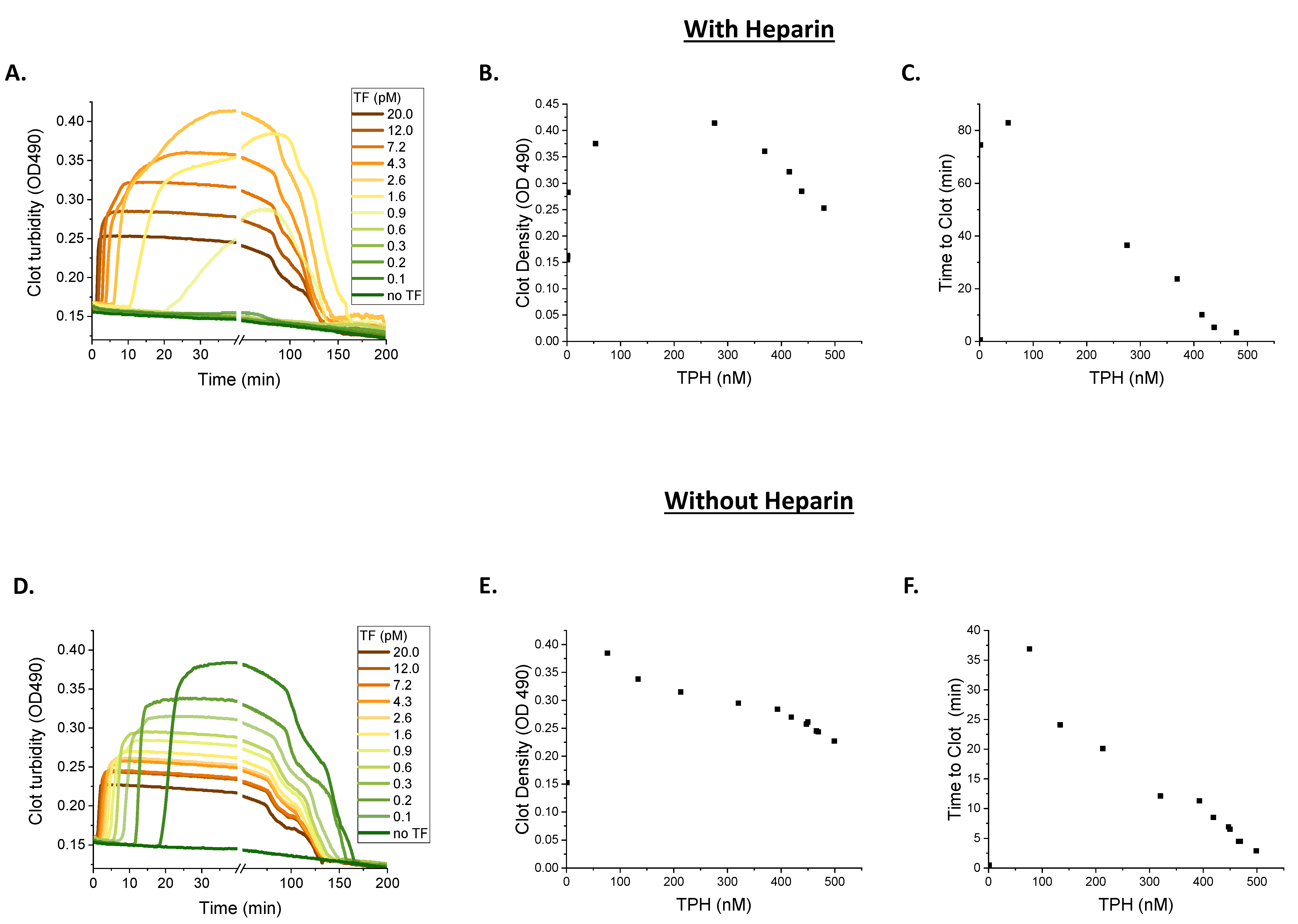

Supplement: Supplementary file 2 — Supplementary Material 2 Fig. S2: Differences in CAT and Origin software correction algorithm. The differences of the CAT and Origin software (CBER) calibration methods may be due to additional CAT-proprietary smoothing algorithms, which are applied to (calibrated) TG curves prior to parameter acquisition. Shown is a representative screenshot of a CAT-calibrated TG curve. Highlighted in red boxes are the reported, calculated peak value of 163.73 nM, even though the TG curve itself shows a peak value of 182.9 nM. [file 12959_2023_549_MOESM2_ESM.tiff]

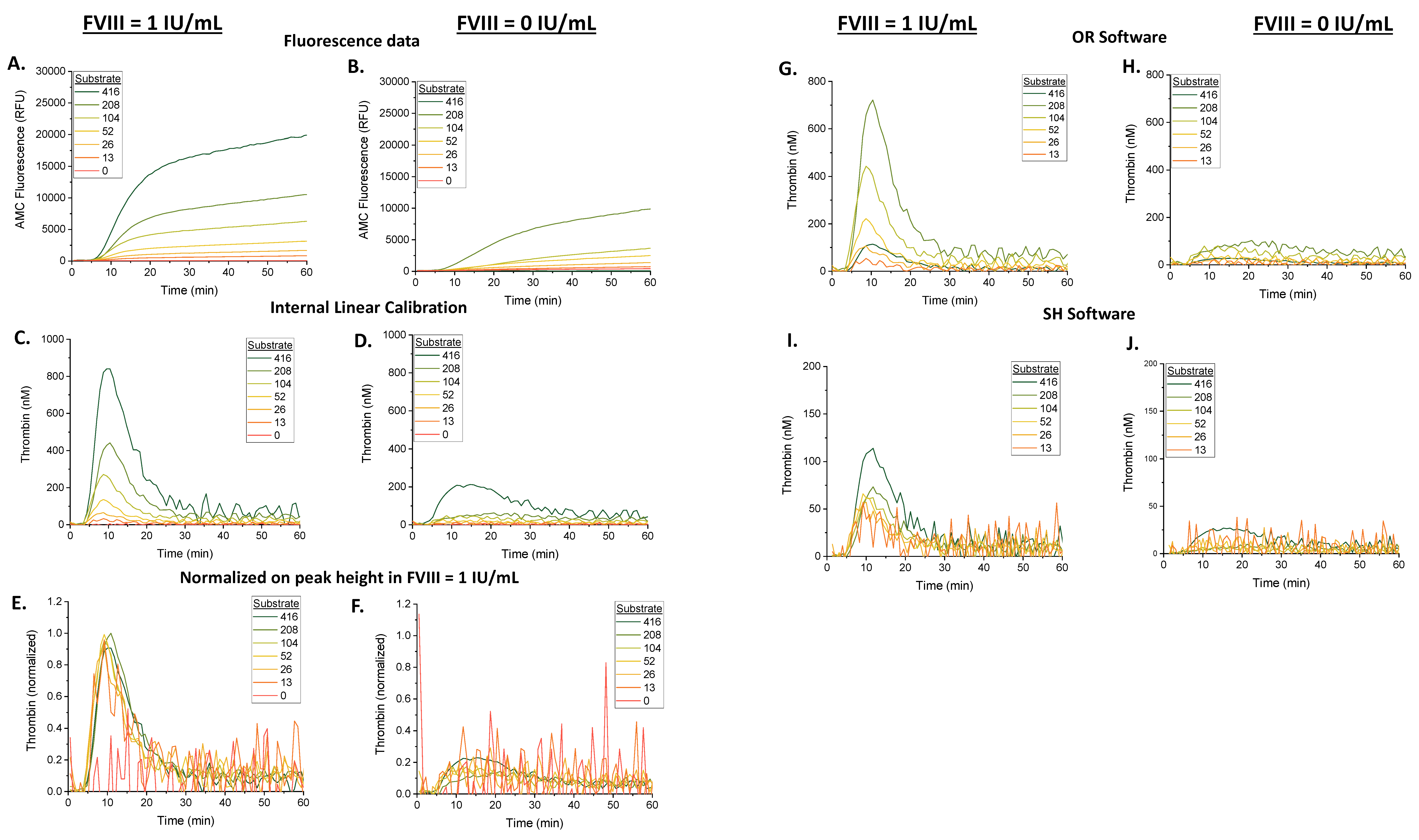

Supplement: Supplementary file 3 — Supplementary Material 3 Fig. S3: AMC and AFC fluorophore emission characteristics. The raw RFUs of the indicated concentrations of (A) AMC and (B) AFC were calculated and showed a proportional increase in fluorescence with increasing concentration. Emission (nm) of AMC was calculated at ~450 nm, whereas AFC was ~490 nm. [file 12959_2023_549_MOESM3_ESM.tiff]

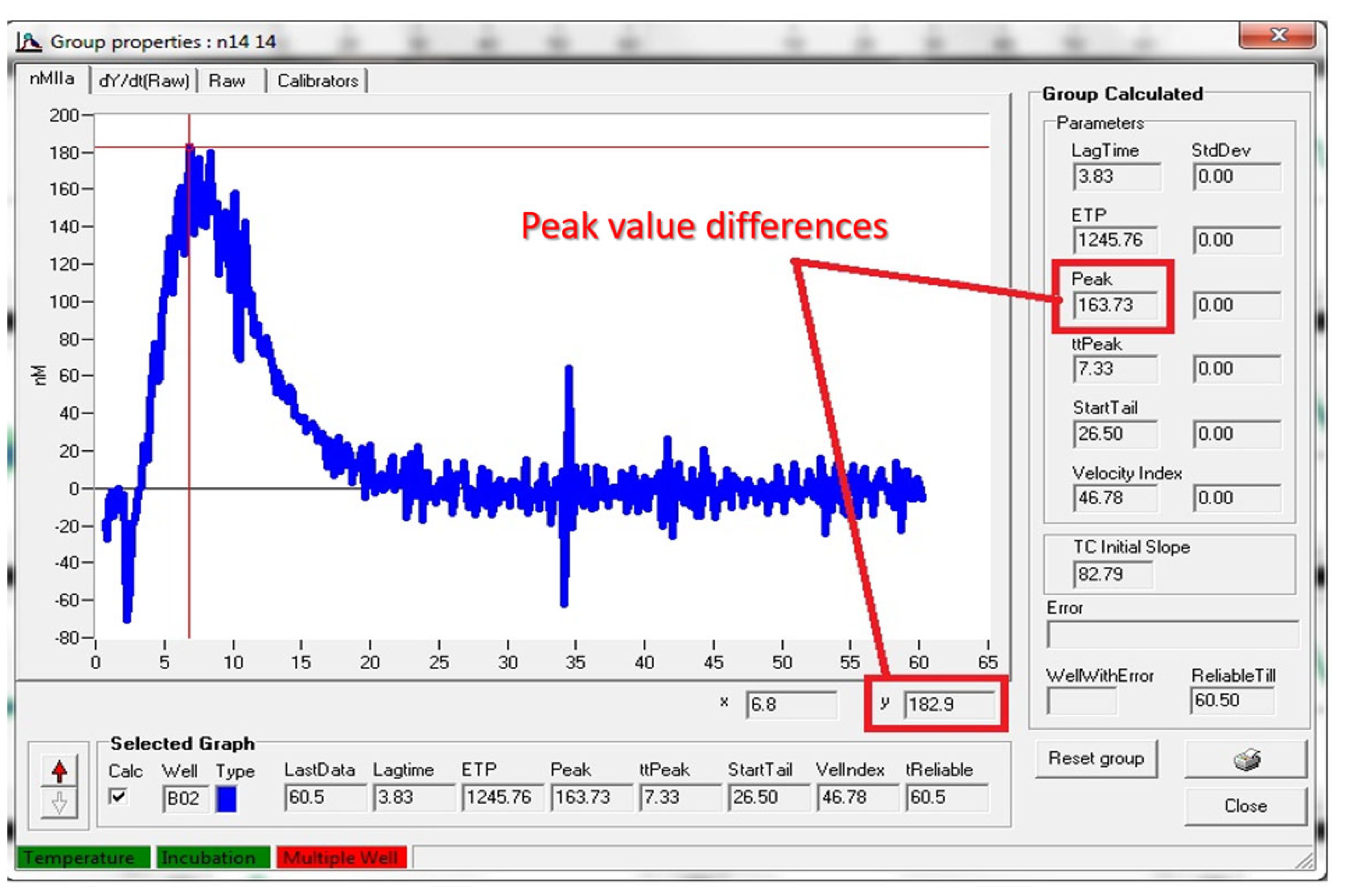

Supplement: Supplementary file 4 — Supplementary Material 4 Fig. S4. Fluorescence of AMC and AFC fluorophores on the CAT microplate reader. The fluorescence of the indicated concentrations of AMC and AFC were measured on the CAT microplate reader. The AFC fluorophore gives a higher signal than that of AMC in CAT instrument demonstrating that this is not suitable for the substrate consumption experiments with added AMC and AFC (Fig. 4). [file 12959_2023_549_MOESM4_ESM.tiff]

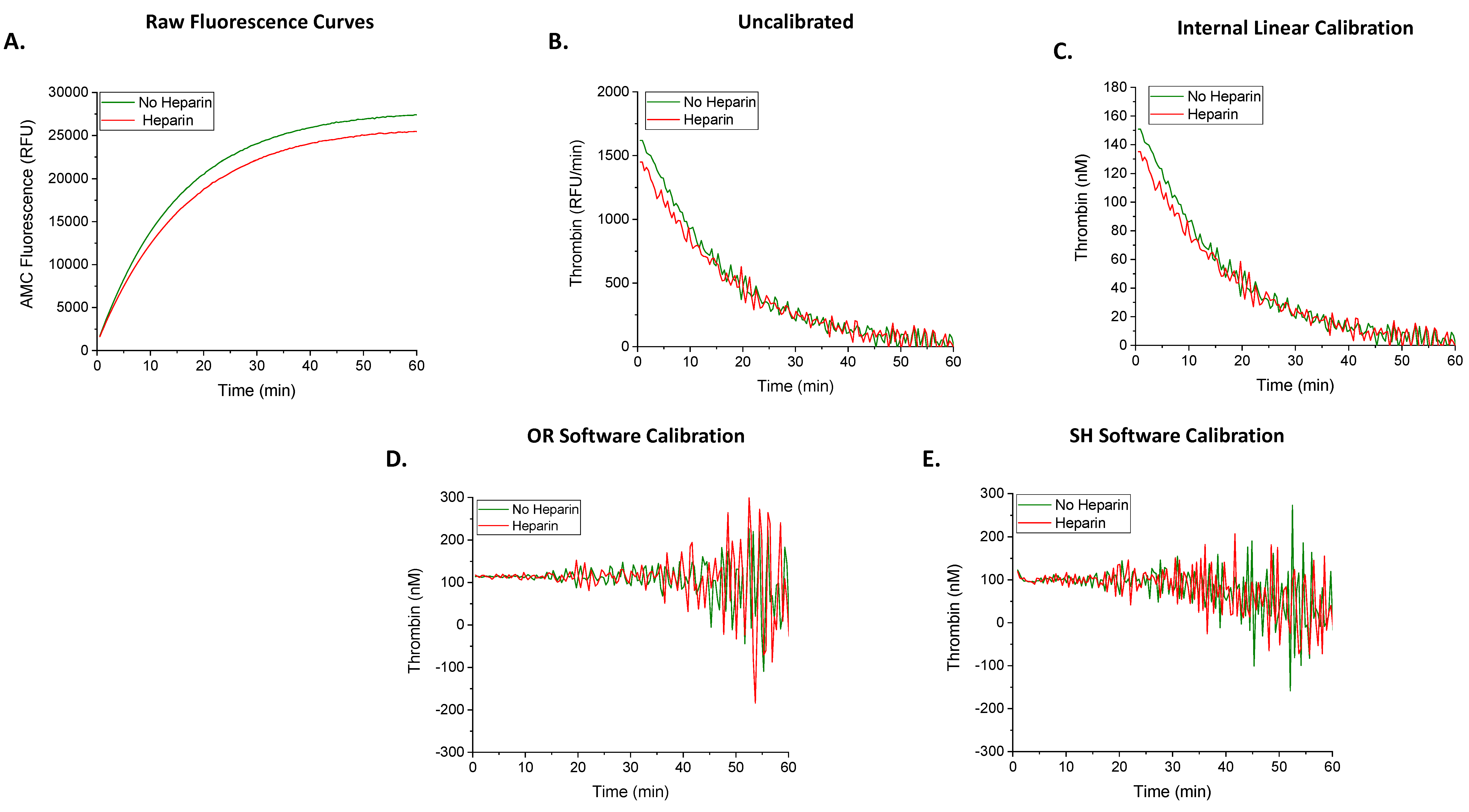

Supplement: Supplementary file 5 — Supplementary Material 5 Fig. S5. Substrate supplementation on FVIII-DP samples. FVIII-DP was supplemented with 1 IU/mL FVIII to normalize plasma, or not, and was subsequently premixed with the indicated concentrations of AMC prior to initiating coagulation with Ca2+. Raw data was produced by the Biotek microplate reader and software and analyzed in several different ways: (A, B) raw AMC fluorescence in relative fluorescent units (RFU), (C, D) Externally calibrated TG curves via a thrombin calibration coefficient (see Materials and Methods), (E, F) Normalized-Uncalibrated curves, (G, H) Calibrated TG curves (via OR software and (I, J) Calibrated TG curves (via SH software). Uncalibrated curve data were produced by differentiating the AMC curves observed in (A, B). Calibrated curves were produced using our in-house OR software, which uses published algorithms similar to CAT calibration, or SH software. Normalized-uncalibrated curves were produced by normalizing each uncalibrated curve pairing of hemophilic and normalized sample (hemophilic plasma supplemented with FVIII) at each pre-spiked AMC concentration against the TPH value of the normalized plasma sample in each pairing. TG was recorded for 40-60 minutes. [file 12959_2023_549_MOESM5_ESM.tiff]

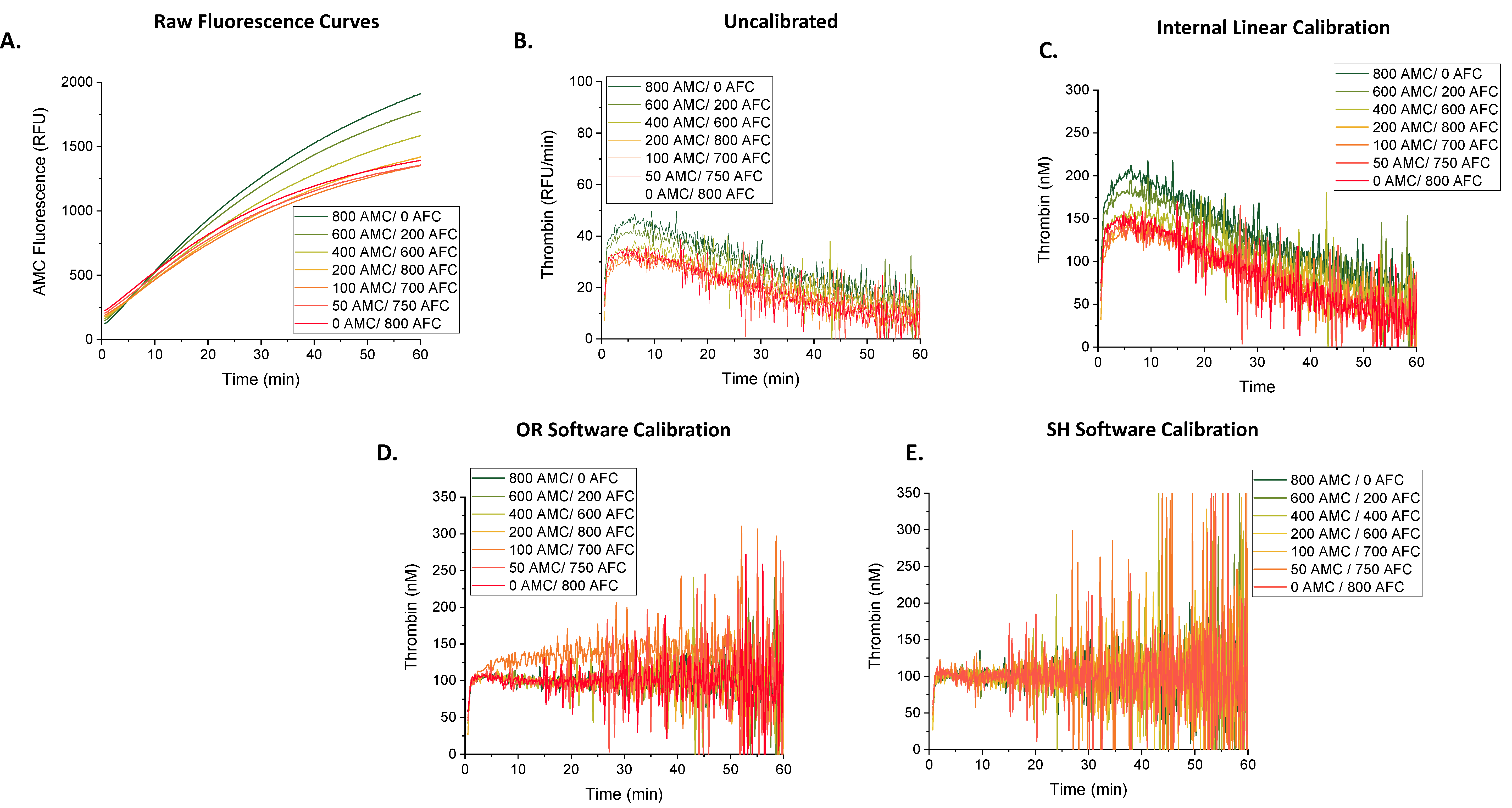

Supplement: Supplementary file 6 — Supplementary Material 6 Fig. S6. Clot formation and lysis in procoagulant samples. ATIII-DP was treated with the indicated concentrations of TF and treated (A) with heparin or (D) without heparin and clot formation was subsequently measured via a fibrin generation (FG) assay (see Materials and Methods). The corresponding correlations between (B, E) clot density (OD 490) vs. TPH (nM) and (C, F) time to clot (min) vs TPH (nM) were plotted. [file 12959_2023_549_MOESM6_ESM.tiff]

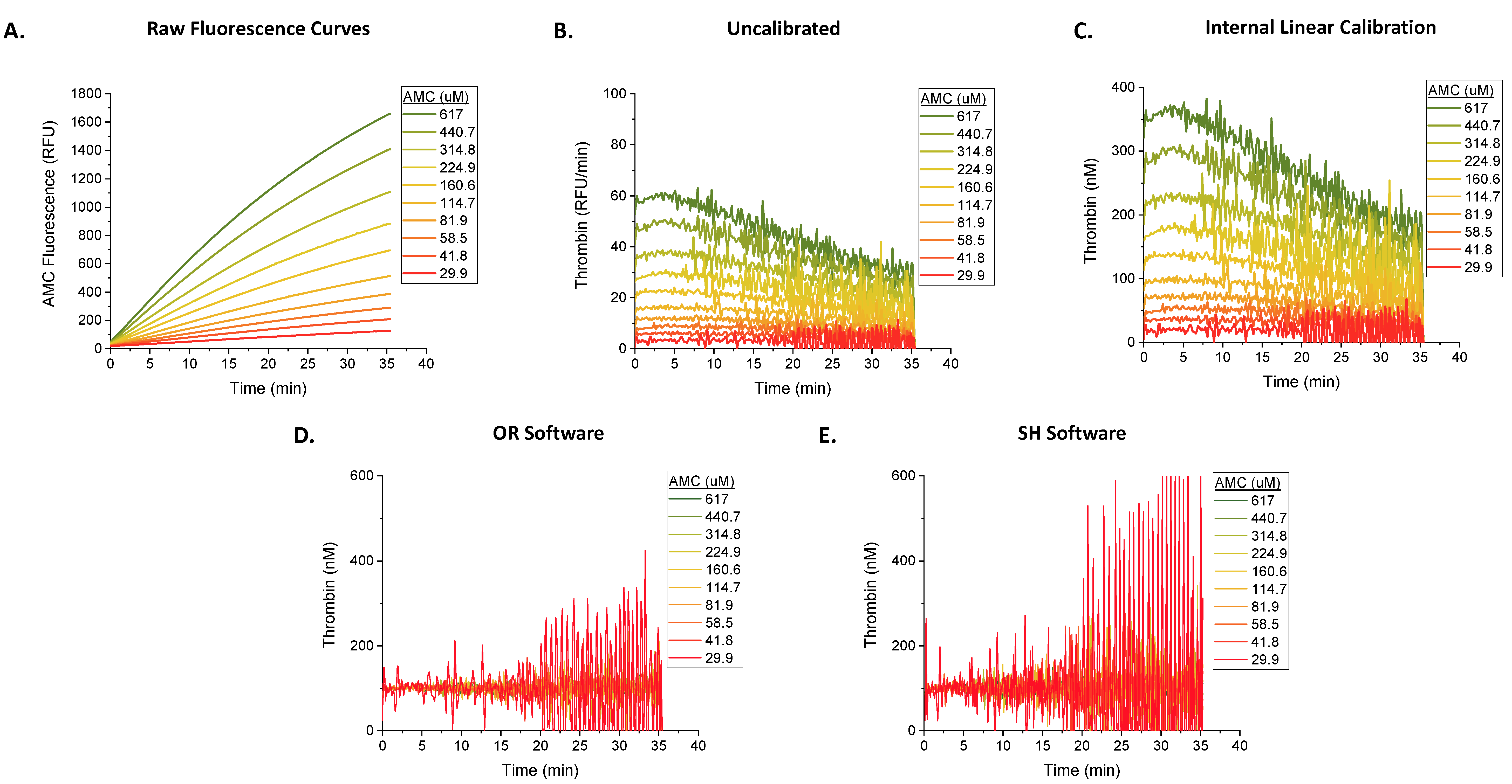

Supplement: Supplementary file 7 — Supplementary Material 7 Fig. S7. Calibration curves for experiments in Fig. 4. Calibration curves for the indicated substrate concentrations are shown: (A) Fluorescence curves, (B) Uncalibrated curves from calibrator wells, (C) calibration curves after Internal Linear calibration, (D) calibration curves after calibration via OR software, and (E) calibration curves after calibration via SH software. [file 12959_2023_549_MOESM7_ESM.tiff]

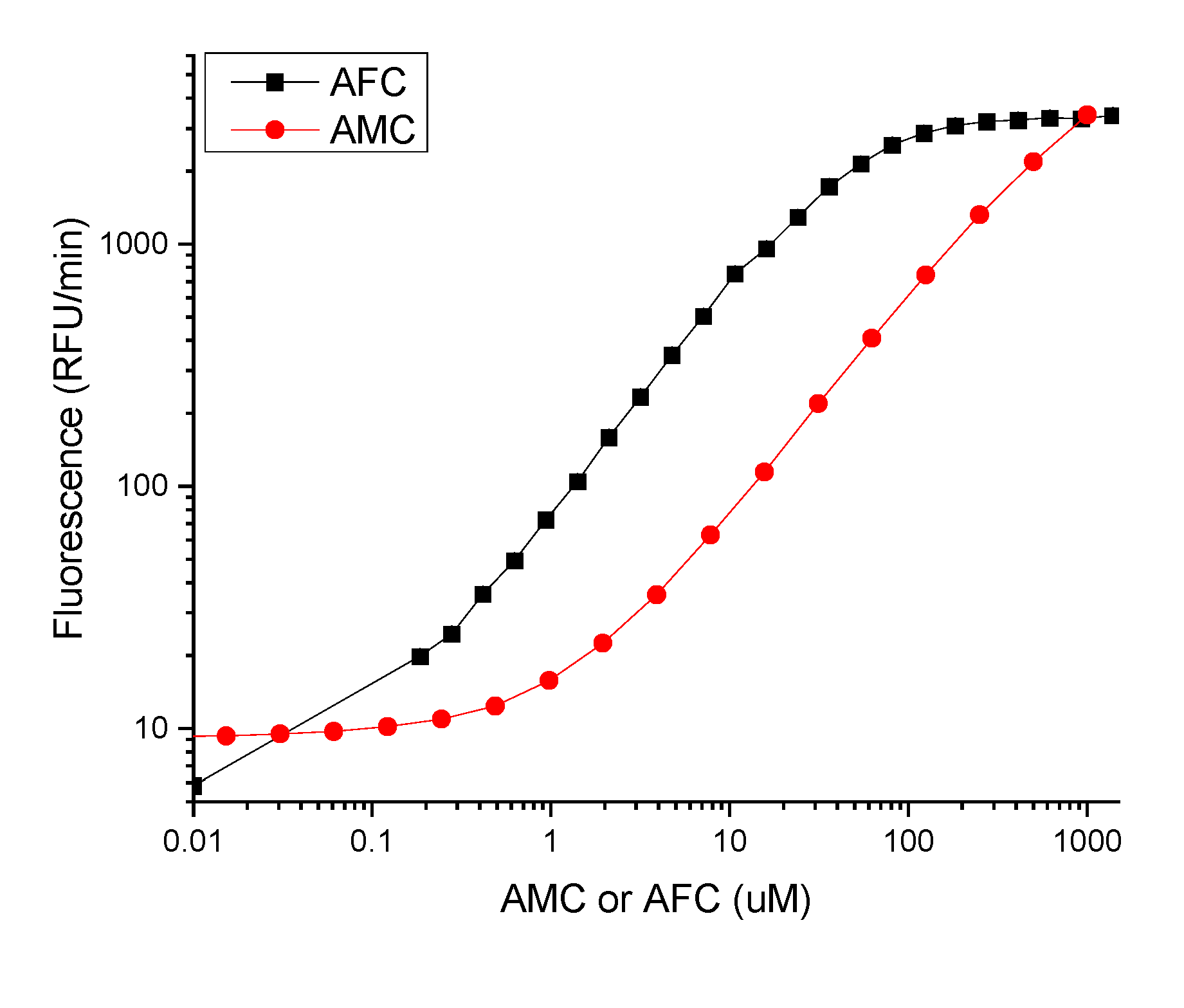

Supplement: Supplementary file 8 — Supplementary Material 8 Fig. S8. Calibration curves for experiments in Fig. 5. Calibration curves for the indicated AMC/AFC substrate concentrations are shown: (A) Fluorescence curves, (B) Uncalibrated curves from calibrator wells, (C) calibration curves after Internal Linear calibration, (D) calibration curves after calibration via OR software, and (E) calibration curves after calibration via SH software. [file 12959_2023_549_MOESM8_ESM.tiff]

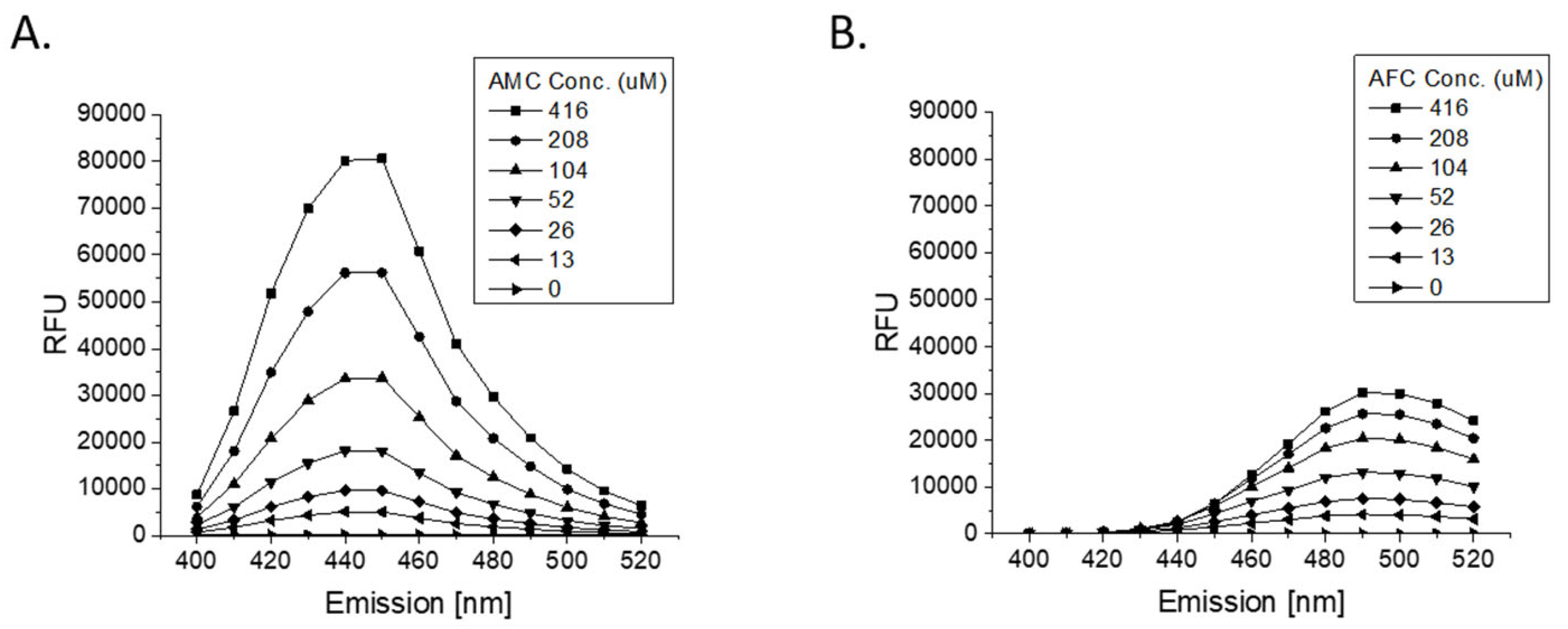

Supplement: Supplementary file 9 — Supplementary Material 9 Fig. S9. Calibration curves for experiments in Fig. 6. Calibration curves in the presence (red curves) or absence (green curves) of heparin: (A) Fluorescence curves, (B) Uncalibrated curves from calibrator wells, (C) calibration curves after Internal Linear calibration, (D) calibration curves after calibration via OR software, and (E) calibration curves after calibration via SH software. [file 12959_2023_549_MOESM9_ESM.tiff]
